# Advantages of cell proliferation and immune regulation in CD146^+^NESTIN^+^ HUMSCs: insights from single-cell RNA sequencing

**DOI:** 10.1093/stmcls/sxae063

**Published:** 2024-10-21

**Authors:** Peng Huang, Xiaofei Qin, Chuiqin Fan, Huifeng Zhong, Manna Wang, Fuyi Chen, Maochuan Liao, Nanpeng Zheng, Hongwu Wang, Bingchun Lin, Lian Ma

**Affiliations:** Shenzhen Maternity and Child Healthcare Hospital, Southern Medical University, Shenzhen, 518028, People’s Republic of China; Department of Hematology and Oncology, Shenzhen Children’s Hospital of China Medical University, Shenzhen, 518038, People’s Republic of China; Shenzhen People’s Hospital, Shenzhen, 518020, People’s Republic of China; Department of Hematology and Oncology, Shenzhen Children’s Hospital of China Medical University, Shenzhen, 518038, People’s Republic of China; Shenzhen Maternity and Child Healthcare Hospital, Southern Medical University, Shenzhen, 518028, People’s Republic of China; Department of Hematology and Oncology, Shenzhen Children’s Hospital of China Medical University, Shenzhen, 518038, People’s Republic of China; Department of Pediatrics, The Second Affiliated Hospital of Shantou University Medical College, Shantou, 515041, People’s Republic of China; Department of Pediatrics, The Second Affiliated Hospital of Shantou University Medical College, Shantou, 515041, People’s Republic of China; Department of Pediatrics, The Second Affiliated Hospital of Shantou University Medical College, Shantou, 515041, People’s Republic of China; Department of Pediatrics, The Second Affiliated Hospital of Shantou University Medical College, Shantou, 515041, People’s Republic of China; Shenzhen Maternity and Child Healthcare Hospital, Southern Medical University, Shenzhen, 518028, People’s Republic of China; Department of Hematology and Oncology, Shenzhen Children’s Hospital of China Medical University, Shenzhen, 518038, People’s Republic of China; Department of Pediatrics, The Second Affiliated Hospital of Shantou University Medical College, Shantou, 515041, People’s Republic of China

**Keywords:** CD146, preterm infants, heterogeneity, HUMSCs, Nestin, single-cell RNA sequencing

## Abstract

The heterogeneity of stem cells is a significant factor inhibiting their clinical application, as different cell subpopulations may exhibit substantial differences in biological functions. We performed single-cell sequencing on human umbilical cord mesenchymal stem cells (HUMSCs) from 3 donors of different gestational ages (22 + 5, 28, and 39 weeks). We also compared the data with single-cell sequencing data from BMSCs from 2 public databases. The content of CD146^+^Nestin^+^ MSCs in preterm HUMSCs (22 + 5W: 30.2%, 28W: 25.8%) was higher than that in full-term HUMSCs (39W: 0.5%) and BMSCs (BMSC1: 0, BMSC2: 0.9%). Cell cycle analysis indicated a higher proportion of cells in the proliferative G2M phase in CD146^+^Nestin^+^ MSCs (40.8%) compared to CD146^+^Nestin^−^ MSCs (20%) and CD146^−^Nestin^−^ MSCs (12.5%). The degree of differentiation assessment suggested that CD146^+^Nestin^+^ MSCs exhibited lower differentiation than other cell subpopulations. Differential gene analysis revealed that CD146^+^Nestin^+^ MSCs overexpressed immune regulation-related factors. GO and KEGG enrichment analysis of modules identified by weighted gene co-expression network analysis suggested enrichment in pathways related to cellular immune regulation, antimicrobial activity, and proliferation. Immune-related gene analysis indicated that CD146^+^Nestin^+^ MSCs exhibited expression of multiple immune-related genes associated with “antimicrobials,” “cytokines,” and “cytokine receptors.” Gene regulatory network analysis revealed high expression of immune-related regulators RELB, GAPB1, and EHF in CD146^+^Nestin^+^ MSCs. Our study provides a single-cell atlas of preterm HUMSCs, demonstrating the expression of CD146^+^Nestin^+^ MSCs across different tissues and confirming their advantages in cellular proliferation, antimicrobial activity, immune regulation, and low differentiation at the RNA level. This contributes valuable insights for the clinical application of HUMSCs.

## Graphical Abstract

**Graphical Abstract:**
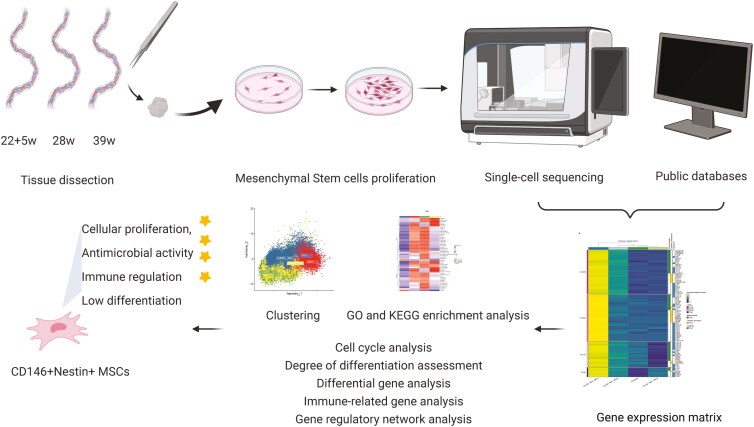

**Significant Statement**

The heterogeneity of stem cells poses a challenge to their clinical application, as the biological functions exhibited in different cell subpopulations may be substantially different. While CD146^+^ and Nestin^+^ cells demonstrate specific functionalities within mesenchymal stem cells (MSCs), their biological characteristics have yet to be fully understood. In this study, we confirmed the advantages of CD146^+^Nestin^+^ MSCs in cellular proliferation, antimicrobial activity, immune regulation, and low differentiation at the RNA level. This provides valuable insights into the clinical application of human umbilical cord MSCs.

## Introduction

According to the standards set by the International Society for Cellular Therapy (ISCT), mesenchymal stem cells (MSCs) can be isolated from various tissues^1^ . However, research indicates that MSCs comprise multiple distinct subpopulations, leading to significant variations in their efficacy. This heterogeneity has resulted in inconclusive therapeutic outcomes in numerous MSC studies for various diseases, with substantial differences in treatment responses observed among individuals.^2^ Therefore, the key to precise MSC therapy lies in the separation of specific functional subpopulations from this heterogeneous cell pool.

Over the years, scientists have delved into the origin of MSCs, often referred to as the “stem cell niche.” Despite ongoing investigations, a definitive consensus has yet to be reached. Tracking murine fetal bone marrow-derived MSCs (BMSCs) has revealed that Nestin^+^ BMSCs remain undifferentiated and retain MSC characteristics from fetal development to birth. In contrast, Nestin^−^ BMSCs differentiate into osteoblasts during embryonic stages and lose MSC properties shortly after birth. Consequently, Nestin^+^ MSCs are suggested to be the progenitor cells of MSCs.^3^ Additionally, some researchers propose that pericytes may serve as precursor cells for MSCs, as CD146^+^ MSCs exhibit characteristics of pericytes while maintaining MSC features.^4^

In our preliminary studies, we obtained Human umbilical cord Wharton’s Jelly derived mesenchymal stem cells (HUMSCs) from patients at various gestational ages (22 + 5, 24 + 4, 25 + 3, 26, 28, 39, and 39 + 2 weeks). Through in vitro CCKB experiments and a series of cell biology assays in coculture with damaged human umbilical vein endothelial cells (HUVECs), we observed that preterm HUMSCs held advantages in cell proliferation and cellular damage repair compared to full-term counterparts.^5^ In this study, we performed single-cell sequencing on umbilical cords from selected cases (22 + 5, 28, and 39 weeks) and integrated single-cell data from MSCs from various tissue sources reported in the literature. Our focus is on analyzing the distribution of CD146^+^ and Nestin^+^ cells in different tissue sources and exploring the functional differences at the RNA level between these cells and other cellular subpopulations.

## Materials and methods

### Isolation and expansion of HUMSCs

This study was approved by the Institutional Review Board of Shenzhen Maternity and Child Healthcare Hospital. HUMSCs were obtained from our laboratory as previously described^6^ and cultured in DMEM/F-12 containing 10% fetal bovine serum. The cells were incubated in a 37°C humidified incubator with 5% CO_2_, and the growth medium was replaced every 2 days.

### Single-cell RNA sequencing

#### Cell preparation

HUMSCs in passage 3 from 3 different gestational ages (22 + 5W, 28W) were used in this study. Cell survival rates were checked and single-cell suspensions with survival rates above 80% were washed and resuspended to a suitable concentration of 700-1200 cells/μL for 10x Genomics Chromium (10X Genome, Single Cell 3ʹ Library & Gel Bead Kit v.3). The system was operated on the machine. GEM creation and thermal cycling: gel bead in emulsion (GEMs) were created for single-cell separation following the number of cells to be harvested. Once the GEMs were formed, they were collected for reverse transcription in a PCR machine for labeling. Post-cycling cleanup & cDNA amplification: the GEMs were treated with oil and the amplified cDNA was purified by magnetic beads. It then underwent cDNA amplification and quality inspection. Library preparation & quantification: The 3ʹ Gene Expression Library was constructed with the quality-qualified cDNA. The library was finally subjected to quantitative analysis following fragmentation, adapter ligation, sample index PCR, and other procedures. Sequencing: The final library pool was sequenced on the Illumina HiSeq3000 using 150-base pair paired-end reads.

### Public database

We downloaded the single-cell transcriptome data of bone marrow-derived MSCs (GSE115149^7^ and GSE162692^8^) from the NCBI public database. Only the gene expression matrix of normal BMSCs was retained, without any modifications. We also downloaded the data of HUMSCs (39W) from Mendeley Data (https://data.mendeley.com/datasets/f4b2ykfv56/2).^9^

### Quality control

The R package Seurat (version 4.0.0)^6,10^ was used for cell quality control and cluster analysis of single-cell transcriptomic data of umbilical cord-derived MSCs and bone marrow-derived MSCs. First, Seurat objects were created for all samples. Second, genes with less than 2000 counts were filtered out, and low-quality cells were removed if mitochondrial genes accounted for more than 10% and red blood cell genes accounted for more than 10%. Finally, all filtered single-cell transcriptome datasets were merged using the merge function, and the distribution of mitochondrial genes and red blood cell genes before and after filtering in each sample was shown using a violin plot.

### Batch effect evaluation and correction

To correct for the sequencing depth of each cell, we normalized and standardized the combined matrix. Next, we performed principal component analysis (PCA) on the top 2,000 genes with high heterogeneity to obtain the first 50 PCA dimensions. We evaluated the contribution of the first 50 PCA dimensions to the data variance using the ElbowPlot function. We then used the R package Harmony (version 1.0) to remove batch effects from the PCA dimensions and obtain the top 50 harmony dimensions. We evaluated the contribution of the top 50 harmony dimensions to the data variance using the ElbowPlot function. By comparing the PCA scatter plot and the Harmony scatter plot, we evaluated the correction of the batch effect.

### Cell subpopulation biological annotation

We used the top 50 harmony dimensions for Uniform Manifold Approximation and Projection (UMAP) dimension reduction. We also used the top 50 harmony dimensions for cluster analysis, with a resolution set to 1.0. Finally, we annotated the cellular subpopulations based on surface markers of MSCs (CD105/ENG^+^, CD90/THY1^+^, CD73/NT5E^+^, CD45/PTPRC^−^, CD34^−^, CD19^−^, HLA^−^DRA^−^, CD11b/ITGAM^−^), fibroblasts (VIM^+^, CD31/PECAM1^−^, CD34^−^, CD45/PTPRC^−^, EPCAM^−^, MYH11^−^) and pericytes (CD146/MCAM^+^, CD31/PECAM1^−^, CD34^−^, CD45/PTPRC^−^).^9^

### Cell cycle inference and cell subtype proportion analysis

We used the CellCycleScoring function in the R package Seurat to evaluate the cell cycle phases of each cell subtype. Then, we showed the proportions of each cell subtype in different cell cycle phases and the proportions of different cell cycle phases in each cell subtype using a bar plot. Meanwhile, we also displayed the proportions of each cell subtype in different samples and the proportions of cells from different sample origins in each cell subtype using a bar plot. To validate the results of the cell cycle analysis, we examined the expression of S-phase representative genes (PCNA and CLSPN) and G2M-phase representative genes (CDK1 and CCNB2) in different cellular subpopulations and visualized them using scatter plots.

### Differential gene analysis

We used the FindMarker function in the R package “Seurat” and set an absolute value of LogFC ≥ 1 and a corrected *P*-value < .05 as the filtering criteria to identify differentially expressed genes with statistical significance in different cell subtypes.

### Single-cell weighted gene co-expression network analysis

To explore the potential biological functions of different cellular subpopulations, we conducted single-cell weighted gene co-expression network analysis (WGCNA) using the R package “hdWGCNA” (version 0.2.18).^7^ Subsequently, we performed Gene Ontology (GO) biological processes (BP) and Kyoto Encyclopedia of Genes and Genomes (KEGG) enrichment analysis on the identified co-expression modules using the R package “clusterProfiler” (version 4.0.0).^11^

### Gene set enrichment analysis

To further validate the results of single-cell WGCNA, we performed gene set enrichment analysis for different cellular subpopulations using the R package “clusterProfiler” (version 4.0.0).^11^ We conducted GO -BP and KEGG gene set enrichment analysis and visualized the enrichment analysis results using volcano plots, Manhattan plots, and heatmaps.

### Analysis of immune-related genes

In the analysis of the single-cell Weighted gene co-expression Network, the CD146^+^ NES^+^ MSCs subpopulation was found to be closely associated with biological processes such as antimicrobial responses and cytokine-mediated signaling pathways. To investigate the potential immune functions of the CD146^+^ NES^+^ MSCs subpopulation, we extracted 1793 immune-related genes from the ImmPort database (https://www.immport.org/shared/genelists, accessed on July 7, 2020^12^; Supplementary Table S4). We then examined the expression of immune-related genes annotated as “antimicrobials,” “cytokines,” and “cytokine receptors” within the CD146^+^ NES^+^ MSCs subpopulation.

### Custom gene set scoring

The multidirectional differentiation, stemness, and immunoregulatory abilities of MSCs have been a focus of basic research. To evaluate the differences in osteogenesis, chondrogenesis, adipogenesis, neurogenesis, angiogenesis, and immune regulation among different MSC subtypes, we collected and curated 6 gene sets from the literature: chondrogenic (SOX5, SOX6, COL10A1, COL11A1, ACAN, and COMP), osteogenic (RUNX2, SP7, ALPL, BGLAP, COL1A1, and OGN), adipogenic (PPARG, CIDEC, ADIPOQ, CD36, FABP4, and AOC3), angiogenesis and neurogenesis (HGF, VEGFA, NGF, FGF2, IGF1, CXCL12, GDNF, BDNF, NGF, IGF1, EGF, and ITGB1).^9^ The genes in these sets have been shown in previous studies to reflect the multidirectional differentiation of MSCs. Therefore, we used the AddModuleScore function in the R package “Seurat” based on these 4 custom gene sets to score different cell subtypes, and visualized their score distribution using a violin plot.

### Degree of differentiation assessment

We used the R package “CytoTRACE” (version 1.8.0)^13^ to infer the differentiation degree of different cell subtypes. We used the iCytoTRACE function in the “CytoTRACE” package to remove the batch effect and evaluate the differentiation degree of different cell subtypes. The CytoTRACE score ranges from 0 to 1, with higher scores indicating a lower differentiation degree. We displayed the CytoTRACE scores of different cell subtypes using a box plot.

### Gene regulatory network analysis

We constructed a gene regulatory network using pySCENIC (version 0.10.3) and identified distinct regulons.^14^ Different regulons consist of various transcription factors and their target genes (refer to Supplementary Table S5). The specificity scores of regulons were computed using the calcRSS function from the R package SCENIC.^14^ Heatmaps were used to visualize the top 3 highly expressed regulons in different MSC subpopulations.

### Building an interactive visualization application

To facilitate the exploration of the single-cell transcriptomic data involved in this study, we created an interactive web application using the R package ShinyCell (version 1.24.1).^15^ This application allows researchers to interactively explore the expression patterns of different genes/regulons in various cellular subpopulations. Additionally, we packaged the application into an executable desktop program using the Electron framework. The desktop executable is compatible with both Windows 32-bit and 64-bit systems and is available for download on the Mendeley database at https://data.mendeley.com/drafts/36tfjc42hm. Researchers can use this tool to visually analyze and interpret the single-cell transcriptomic data, enhancing their ability to delve into the intricacies of gene and regulon expression across different cell subpopulations.

### Cell sorting

Flow cytometry was used to sort CD146^+^NES^+^ cells. The cells were first fixed and permeabilized to allow intracellular staining through FoxP3/Transcription Factor Fixation (eBioscience , Cat# 00-5521-00). For the detection of Nestin, cells were incubated with a primary antibody, Nestin (rabbit anti-human, polyclonal, Sigma-Aldrich, Cat# N5413), followed by a secondary antibody, Goat Anti-Rabbit conjugated with BV421 (BD Pharmingen, Cat# 565014). For CD146, cells were directly stained with a conjugated antibody, CD146 (mouse anti-human, Alexa Fluor 647, BD Pharmingen, Cat# 563619). After staining, cells were washed, resuspended in a buffer, and analyzed on a flow cytometer with appropriate controls to ensure specificity and accuracy.

### Quantitative reverse transcription-polymerase chain reaction

Cells were lysed using TRIzol reagent to extract total RNA. The RNA was purified by chloroform extraction and isopropanol precipitation, followed by washing with 75% ethanol. RNA concentration and purity were assessed using a spectrophotometer. Reverse transcription was performed using a cDNA synthesis kit, where one µg of RNA was converted into cDNA according to the manufacturer’s protocol. qRT-PCR was carried out using SYBR Green Master Mix on a real-time PCR system. Specific primers for target genes and a housekeeping gene were used. The cycling conditions included initial denaturation, followed by 40 cycles of denaturation, annealing, and extension. Relative gene expression levels were calculated using the 2^−ΔΔCt^ method, with normalization to the housekeeping gene. The primer sequence is as follows: BUB3 (forward: GGACCCATGATGCCCCTATC, upstream: CCCAGCATTACAAGGAGTTCTG), CCL2 (forward: CAGCCAGATGCAATCAATGCC, upstream: TGGAATCCTGAACCCACTTCT), CDK1 (forward: AAACTACAGGTCAAGTGGTAGCC, upstream: TCCTGCATAAGCACATCCTGA), CXCL1 (forward: CCAAACCGAAGTCATAGCCAC, upstream: AGGAACAGCCACCAGTGAG), CXCL3 (forward: CGCCCAAACCGAAGTCATAG, upstream: GCTCCCCTTGTTCAGTATCTTTT), CXCL5 (forward: AGCTGCGTTGCGTTTGTTTAC, upstream: TGGCGAACACTTGCAGATTAC), FAU (forward: CCCGGAAGATCAAGTCGTGC, upstream: CACCTTAGGAGTCTGACCTCTC), GADD45A (forward: GAGAGCAGAAGACCGAAAGGA, upstream: CACAACACCACGTTATCGGG), ORC6 (forward: ACAAGGAGACATATCAGAGCTGT, upstream: AGTGGCCTGGATAAGTCAAGAT), RELB (forward: TTCCGAGCCCGTCTATGACAA, upstream: CGTCTTGAACACAATGGCAATC), RPL30 (forward: AAGGCAAAGCGAAATTGGTCA, upstream: TGCCACTGTAGTGATGGACAC), and RPL39 (forward: TGCTGTCTGAAGGTCACGAT, upstream: AATCCAGCCAACCAACGTGT).

### Western blotting

Cells were lysed in RIPA buffer containing protease inhibitors, and the lysates were clarified by centrifugation. Protein concentration was determined using a BCA assay. Equal amounts of protein samples were separated by SDS-PAGE and transferred onto PVDF membranes. The membranes were blocked with 5% nonfat milk in TBST for 1 hour at room temperature. After blocking, the membranes were incubated overnight at 4 °C with the following primary antibodies from Proteintech: RELB (Cat# 80906-1-RR), FAU (Cat# 13581-1-AP), CXCL5 (Cat# 10809-1-AP), and β-actin (Cat# 66009-1-Ig). The membranes were then washed and incubated with the appropriate secondary antibodies: HRP-conjugated Goat Anti-Mouse IgG (Cat# SA00001-1) or Biotin-conjugated Goat Anti-Rabbit IgG (Cat# SA00004-2). Protein bands were visualized using chemiluminescence (ECL) and detected on a digital imaging system.

## Results

### Quality control

Seurat objects included 5 MSC samples: 2 BMSCs (BMSC1 and BMSC2) and 3 HUMSCs (22 + 5W, 28W, and 39W). The violin plot was used to show the distribution of nFeature RNA, nCount_RNA, percent.mt, percent.HB, and percent.ribo of 5 samples before and after quality control (Supplementary Figures S1 and S2). Quality control was performed using the criteria of nFeature RNA >= 2000 & percent.mt <= 10 & percent.HB <= 10. This resulted in the identification of 32 738 UMI in BMSC1, 33 694 in BMSC2, and 33 538 in each of the HUMSCs (22 + 5W, 28W, and 39W).

### Integration analysis

In our study, Harmony was used to eliminate batch effects and integrate single-cell data, ensuring that subsequent analyses only accounted for biological differences (Supplementary Figure S3). The reduction plot contrasting PCA dimensionality and Harmony dimensionality displayed a significant overlap among MSCs derived from the same tissues, specifically the umbilical cord and bone marrow, and partial overlap among different tissues (Supplementary Figure S3). These results indicated an effective correction of batch effects from distinct datasets, leading to the identification of biological differences between MSCs obtained from varying tissues. Furthermore, to identify major cell subsets, we used the first 50 Harmony dimensions for UMAP, with a clustering resolution of 0.5, successfully assigning 17 distinct cell subsets labeled as C0-C16.

### Notes on the biology of cell subsets

**Figure 1.**
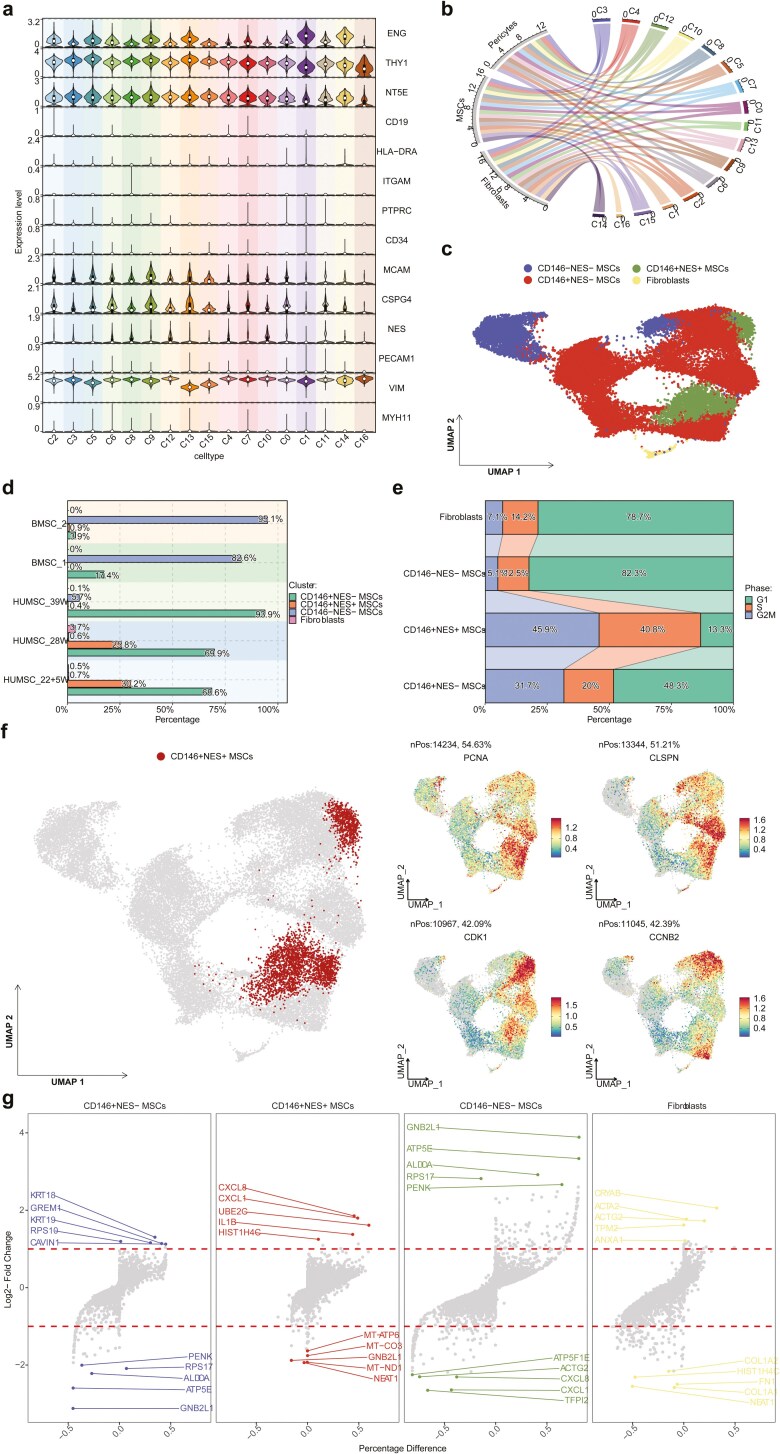
Analysis of single-cell transcriptome data. (A) Violin plots display the expression patterns of surface markers in different clusters, including MSCs (CD105/ENG^+^, CD90/THY1^+^, CD73/NT5E^+^, CD45/PTPRC^−^, CD34^−^, CD19^−^, HLA^−^DRA^−^, and CD11b/ITGAM^−^), fibroblasts (VIM^+^, CD31/PECAM1^−^, CD34^−^, CD45/PTPRC^−^, EPCAM^−^, and MYH11^−^), and pericytes (CD146/MCAM^+^, CD31/PECAM1^−^, CD34^−^, and CD45/PTPRC^−^). (B) Chord plots illustrate the phenotypic overlap between MSCs, fibroblasts, and pericytes in different clusters. (C) Scatter plots demonstrate cell annotations on UMAP dimensions. (D) Bar charts depict the proportions of different MSC subpopulations from various sample sources. (E) Bar charts show the proportions of different cell cycle phases within different MSC subpopulations. (F) The large scatter plot on the left highlights CD146^+^NES^+^ cells, while the smaller scatter plot on the right displays the expression of S-phase representative genes (PCNA and CLSPN) and G2M-phase representative genes (CDK1 and CCNB2) in different MSC subpopulations. (G) Volcano plots exhibit the top 5 differentially expressed genes in different MSC subpopulations.

We annotated the cellular subpopulations based on surface markers of MSCs (CD105/ENG^+^, CD90/THY1^+^, CD73/NT5E^+^, CD45/PTPRC^−^, CD34^−^, CD19^−^, HLA^−^DRA^−^, and CD11b/ITGAM^−^), fibroblasts (VIM^+^, CD31/PECAM1^−^, CD34^−^, CD45/PTPRC^−^, EPCAM^−^, MYH11^−^), and pericytes (CD146/MCAM^+^, CD31/PECAM1^−^, CD34^−^, CD45/PTPRC^−^). All cells expressed the fibroblast marker (VIM^+^) in all subsets, but not the endothelial cell marker CD31, hematopoietic cell marker CD34, immune cell marker CD45, epithelial marker EPCAM, and smooth muscle marker MYH11 (Figure 1A, B). All but the C16 subsets displayed a phenotype almost consistent with MSCs CD105^+^, CD90^+^, CD73^+^, CD45^−^, CD34^−^, CD19^−^, HLA^−^DRA^−^, annnnnCD11b^−^), confirming that the HUMSC isolated and cultured in our study met the MSC criteria. There were no significant differences in the MSC phenotype among cells derived from different sources. C1, C11, and C14 had no CD146^+^ cells. The majority of other cell subsets (C2, C3, C5, C6, C9, C13, C15, C4, C7, and C0) showed the expression, albeit some cell subsets at a low level, of CD146 and CSPG4, the pericyte-specific gene, indicating the presence of a certain proportion of pericytes or pericyte-related characteristics (Figure 1A, B). We also observed Nestin^+^ cells in C8, C10, and C12 with relatively high expression levels, particularly in C10 and C12. Thus, we identified 4 cell types: Fibroblasts, CD146^−^ NES^−^ MSCs, CD146^+^ NES^+^ MSCs, and CD146^+^ NES^−^ MSCs (Figure 1C). The proportion of CD146^+^ NES^+^ MSCs in preterm HUMSCs varies with gestational age, with percentages of 30.2% at 22 weeks and 25.8% at 28 weeks. Notably, as gestational age decreases, the proportion of CD146^+^ NES^+^ MSCs tends to increase. In full-term HUMSCs and BMSCs, the proportion of CD146^+^ NES^+^ MSCs is minimal. Specifically, in full-term HUMSCs, the majority are CD146^+^ NES^−^ MSCs, accounting for 93.9% of the population, while in BMSCs, the predominant population is CD146^−^ NES^−^ MSCs, representing 95.1% and 82.6% of the total, respectively (Figure 1D).

### Cell cycle inference and cell subtype proportion analysis

We used the CellCycleScoring function in the R package Seurat to evaluate the cell cycle phases of each cell subtype. We observed that G2M phase cells accounted for 45.9% of CD146^+^ NES^+^ MSCs, 31.7% of CD146^+^ NES^−^ MSCs, 5.1% of CD146^−^ NES^−^ MSCs, and 7.1% of fibroblasts (Figure 1E). S-phase cells comprised 40.8% of CD146^+^ NES^+^ MSCs, 20% of CD146^+^ NES^−^ MSCs, 12.5% of CD146^−^ NES^−^ MSCs, and 14.2% of fibroblasts (Figure 1E). To validate the results of the cell cycle analysis, we performed scatter plots visualizing the expression of S-phase representative genes (PCNA and CLSPN) and G2M-phase representative genes (CDK1 and CCNB2) in different cell subpopulations (Figure 1F). We discovered that PCNA, CLSPN, CDK1, and CCNB2 exhibited higher expression in both CD146^+^ NES^+^ MSCs and CD146^+^ NES^−^ MSCs. Furthermore, the expression of CDK1 and CCNB2 was higher in CD146^+^ NES^+^ MSCs compared to CD146^+^ NES^−^ MSCs, consistent with the cell cycle inference results.

### Differential gene analysis

We used the FindMarker function in the R package “Seurat” and set an absolute value of LogFC ≥ 1 and a corrected *P*-value < .05 as the filtering criteria to identify differentially expressed genes with statistical significance in different cell subtypes. We identified 30, 66, 213, and 121 differentially expressed genes in CD146^+^ NES^+^ MSCs, CD146^+^ NES^−^ MSCs, CD146^−^ NES^−^ MSCs, and fibroblasts, respectively (Supplementary Table S1). The volcano plot displays the top 5 upregulated and downregulated differentially expressed genes in each subgroup (Figure 1G).

### Single-cell weighted gene co-expression network analysis

**Figure 2.**
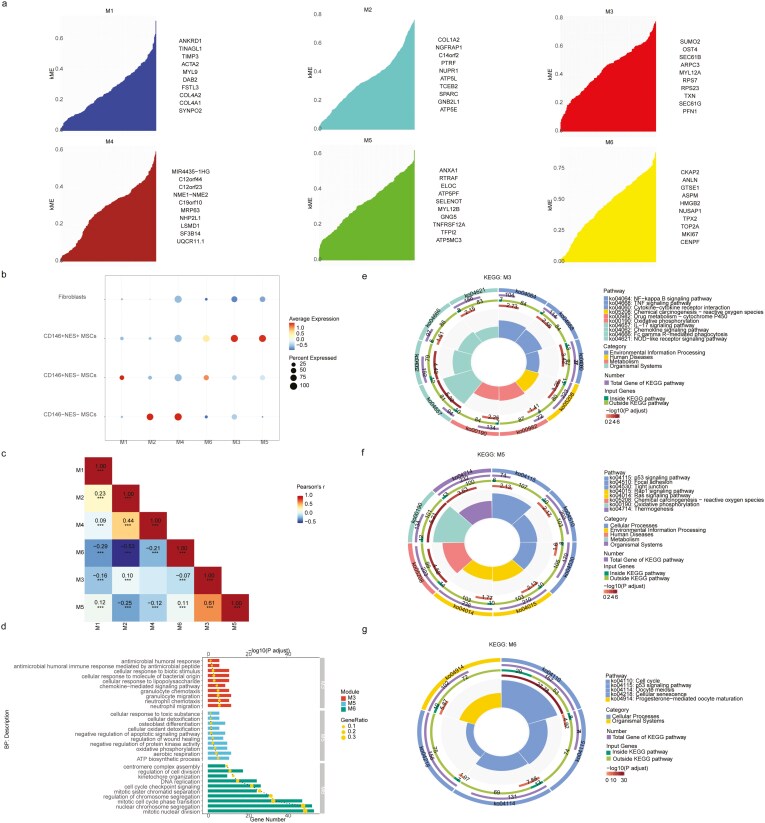
Analysis of single-cell weighted gene co-expression network. (A) Identification of co-expression modules and the top 10 genes in each module. (B) Bubble plots depict the expression of different modules in various MSC subpopulations. (C) On-diagonal heatmaps show the correlation between different modules. (D) Bar charts illustrate statistically significant Gene Ontology Biological Process (GO-BP) terms enriched in modules M3, M5, and M6. (E-G) Circular plots display statistically significant KEGG pathway enrichment for modules M3, M5, and M6. Each circular plot consists of 5 rings. The outermost ring includes KEGG pathway identifiers and functional categories. The second - outermost ring represents the total number of genes in each KEGG pathway. The third ring represents genes enriched and not enriched in the input genes for that KEGG pathway. The fourth ring represents the *P*-value for each KEGG pathway. The innermost ring shows the GeneRatio ranking for KEGG pathways.

We conducted single-cell weighted gene co-expression network analysis using the R package hdWGCNA and performed gene set enrichment analysis on the identified co-expression modules to characterize the potential biological functions of different cell subpopulations. We constructed a gene co-expression network and identified 7 modules through hierarchical clustering (Supplementary Figure S5). Aside from the gray module, which represents a collection of genes without co-expression relationships and was omitted in downstream analysis, all other modules consist of genes with co-expression relationships (Supplementary Table S2). We computed the connectivity of each module and displayed the top 10 highly connected genes (Figure 2A). Genes with high connectivity within each module are considered hub genes. Subsequently, we calculated the harmonized module eigengenes, which quantify the overall expression of each module. We visualized the expression of different modules in various cell subpopulations using a bubble plot and observed that M6, M3, and M5 were highly expressed in CD146^+^ NES^+^ MSCs, with M6 also exhibiting high expression in CD146^+^ NES^−^ MSCs (Figure 2B). To validate the independence of the modules from each other, we calculated the inter-module correlations and visualized them using a diagonal heatmap (Figure 2C). We found that M6 had minimal correlations with M3 and M5 (*r* = −0.07, *r* = 0.11), while M3 exhibited a positive correlation with M5 (*r* = 0.61). We performed gene set enrichment analysis on the genes within M6, M3, and M5 separately using the R package clusterProfiler (Figure 2D-G; Supplementary Table S2). In GO-BP enrichment analysis: M3 was enriched in antimicrobial-related biological processes such as “cellular response to lipopolysaccharide,” “cellular response to molecule of bacterial origin,” “cellular response to biotic stimulus,” “antimicrobial humoral immune response mediated by antimicrobial peptide,” and “antimicrobial humoral response.” It also enriched chemotaxis-related processes, including “neutrophil migration,” “neutrophil chemotaxis,” “granulocyte migration,” “granulocyte chemotaxis,” and “chemokine-mediated signaling pathway.” M5 was enriched in energy metabolism-related biological processes, such as “ATP biosynthetic process,” “aerobic respiration,” and “oxidative phosphorylation,” as well as oxidation-related processes like “cellular oxidant detoxification,” “cellular detoxification,” and “cellular response to toxic substance.” M6 was enriched in cell proliferation-related biological processes, including “mitotic nuclear division,” “nuclear chromosome segregation,” “mitotic cell cycle phase transition,” “regulation of chromosome segregation,” “mitotic sister chromatid separation,” “cell cycle checkpoint signaling,” “DNA replication,” “kinetochore organization,” “regulation of cell division,” and “centromere complex assembly.” In KEGG enrichment analysis: M3 was enriched in chemotaxis-related signaling pathways like “chemokine signaling pathway” and “cytokine-cytokine receptor interaction.” M5 was enriched in oxidative phosphorylation-related pathways, specifically “oxidative phosphorylation.” M6 was enriched in cell proliferation-related signaling pathways, including “cell cycle” and “p53 signaling pathway.”

### Gene set enrichment analysis

**Figure 3.**
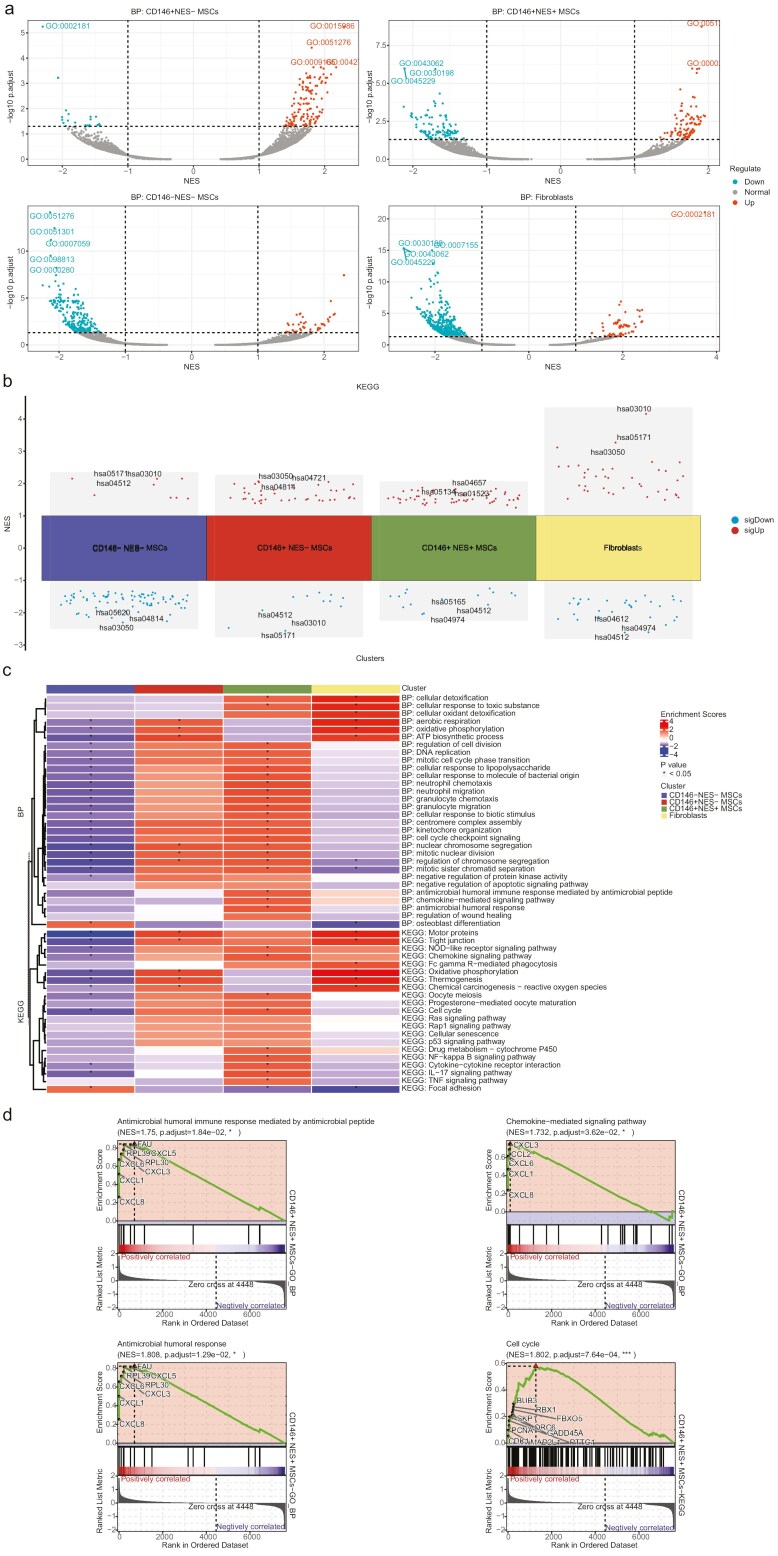
Gene set enrichment analysis. (A) Volcano plot displays the top 5 significantly enriched Gene Ontology Biological Process (GO-BP) terms with statistically significant expression differences (absolute values) in different MSC subpopulations. (B) Manhattan plot illustrates the top 5 significantly enriched Kyoto Encyclopedia of Genes and Genomes (KEGG) pathways with statistically significant expression differences (absolute values) in different MSC subpopulations. (C) Heatmap shows the enrichment scores and *P*-values for selected GO-BP terms and KEGG pathways in different MSC subpopulations. (D) Gene Set Enrichment Analysis (GSEA) plots for the “antimicrobial humoral immune response mediated by antimicrobial peptide,” “chemokine-mediated signaling pathway,” “antimicrobial humoral response,” and “cell cycle” pathways.

To further validate the results of the single-cell weighted gene co-expression network analysis, we conducted gene set enrichment analysis (GSEA) for different cell subpopulations using the “clusterProfiler” package in R (Figure 3A, B; Supplementary Table S3). In the GO-BP enrichment analysis, CD146^+^ NES^+^ MSCs were enriched in antimicrobial-related biological processes such as “cellular response to lipopolysaccharide,” “cellular response to molecule of bacterial origin,” “cellular response to biotic stimulus,” “antimicrobial humoral immune response mediated by antimicrobial peptide,” “antimicrobial humoral response,” as well as chemotaxis-related processes like “neutrophil migration,” “neutrophil chemotaxis,” “granulocyte migration,” “granulocyte chemotaxis,” and “chemokine-mediated signaling pathway.” Additionally, they were enriched in cell proliferation-related biological processes such as “mitotic nuclear division,” “nuclear chromosome segregation,” “mitotic cell cycle phase transition,” “regulation of chromosome segregation,” “mitotic sister chromatid separation,” “cell cycle checkpoint signaling,” “DNA replication,” “kinetochore organization,” “regulation of cell division,” and “centromere complex assembly” (Figure 3C). CD146^+^ NES^−^ MSCs also showed enrichment in cell proliferation-related processes, including “mitotic nuclear division,” “nuclear chromosome segregation,” and “regulation of chromosome segregation.” In KEGG enrichment analysis, CD146^+^ NES^+^ MSCs were enriched in signaling pathways such as the “chemokine signaling pathway,” “cytokine-cytokine receptor interaction,” and “cell cycle” (Figure 3C). Finally, we highlighted the specific enrichment of 4 items: “antimicrobial humoral immune response mediated by antimicrobial peptide,” “chemokine-mediated signaling pathway,” “antimicrobial humoral response,” and “cell cycle” using GSEA plots (Figure 3D).

### Analysis of immune-related genes

**Figure 4.**
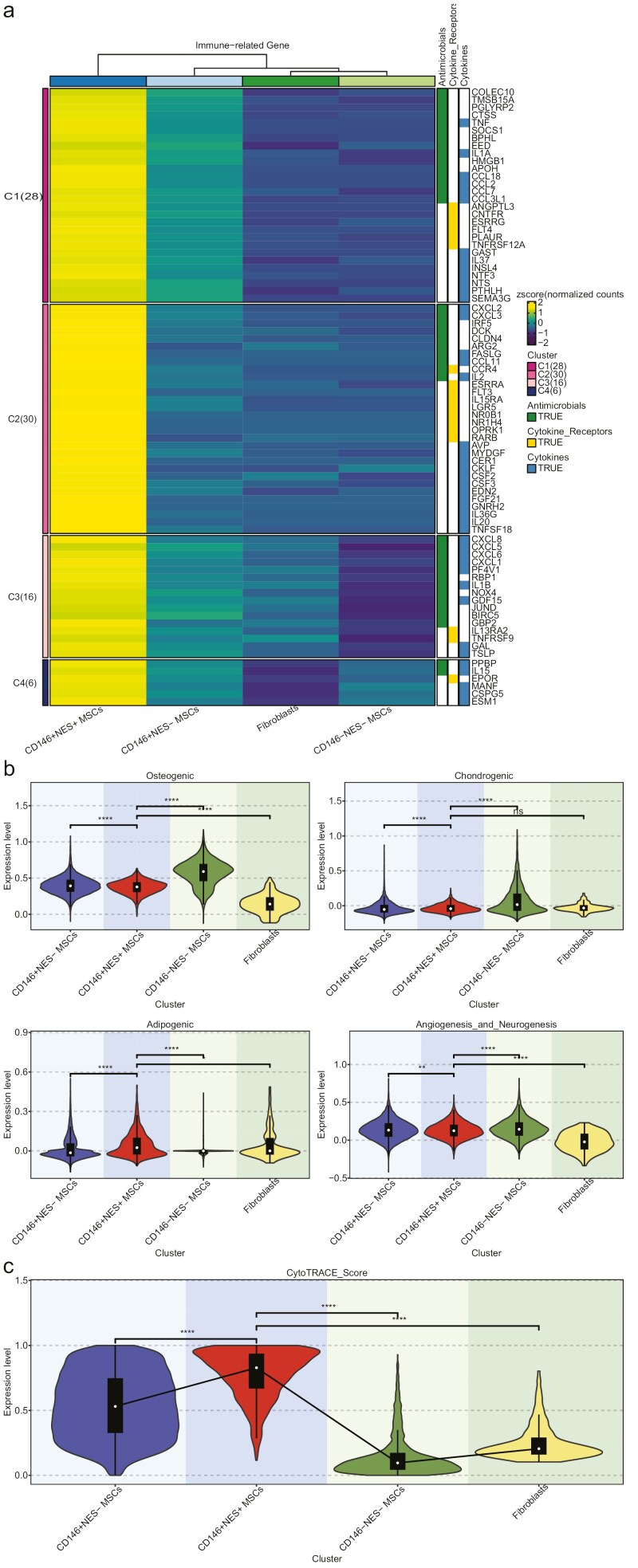
Immunoregulatory gene analysis and lineage inference. (A) The heatmap illustrates the expression profiles of immune-related genes annotated as “antimicrobials,” “cytokines,” or “cytokine receptors” in the CD146^+^ NES^+^ MSCs subpopulation. (B) Violin plots depict the differentiation capacities of distinct MSC subpopulations toward Osteogenic, Chondrogenic, Adipogenic, and Angiogenesis_and_Neurogenesis. (C) Violin plots depict the differentiation extents of diverse MSC subpopulations. A higher CytoTRACE score approaching 1.0 indicates a lower level of differentiation, while a score closer to 0.0 signifies a higher level of differentiation.

In the analysis of the single-cell weighted gene co-expression network, the CD146^+^ NES^+^ MSCs subpopulation was found to be closely associated with biological processes related to antimicrobial and cytokine-mediated signaling pathways. To analyze the potential immune functions of the CD146^+^ NES^+^ MSCs subpopulation, we extracted 1793 immune-related genes from the ImmPort database (Supplementary Table S4) and examined the expression of immune-related genes annotated as “antimicrobials,” “cytokines,” and “cytokine receptors” in different cell subpopulations. Specifically, we focused on the expression of 39 antimicrobials-related genes, 41 cytokines-related genes, and 18 cytokine receptors-related genes in the CD146^+^ NES^+^ MSCs subpopulation (Figure 4A).

### Evaluation of differentiation capacity and differentiation degree

We assessed the functional attributes of cellular subsets by examining their potential for differentiation into osteoblasts, adipocytes, and chondrogenic cells, and their capacity to promote angiogenesis and neurogenesis. We observed that the CD146^+^ NES^+^ MSCs subpopulation exhibited a strong adipogenic potential, whereas the CD146^+^ NES^−^ MSCs subpopulation displayed robust osteogenic and chondrogenic capabilities. Conversely, fibroblasts exhibited comparatively lower abilities in osteogenesis, angiogenesis, and neurogenesis (as illustrated in Figure 4B). To quantify the differentiation degree among different cell subtypes, we used the R package “CytoTRACE.” The CytoTRACE score, which ranges from 0 to 1, was used for this purpose, with higher scores indicating a lower degree of differentiation. Our analysis revealed that both the CD146^+^ NES^+^ MSCs and CD146^+^ NES^−^ MSCs subpopulations exhibited lower degrees of differentiation, while the CD146^+^ NES^+^ MSCs and CD146^+^ NES^+^ MSCs subpopulations displayed higher degrees of differentiation (as shown in Figure 4C). Notably, when comparing the CD146^+^ NES^+^ MSCs subpopulation to the CD146^+^ NES^−^ MSCs subpopulation, the former appeared to exhibit a more primitive state in terms of differentiation degree (Figure 4C).

### Gene regulatory network analysis

**Figure 5.**
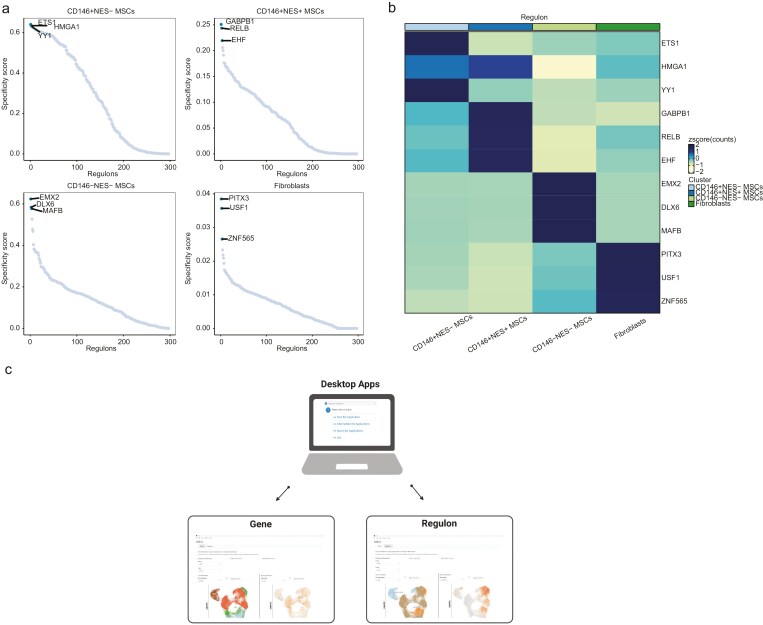
Gene regulatory network analysis and desktop executable program construction. (A) The top 3 highly expressed regulators in distinct MSC subpopulations. (B) The heatmap displays the expression patterns of the top 3 highly expressed regulators in different MSC subpopulations. (C) An overview diagram of the desktop executable program, enabling researchers to query gene and regulator expression in different MSC subpopulations.

We used pySCENIC to construct gene regulatory networks and identified a total of 297 regulators. Regulators with fewer than 10 target genes were filtered out, resulting in 214 regulators (refer to Supplementary Table S5). We computed the specificity scores of these regulators and visualized the top 3 highly expressed regulators in different MSC subpopulations (Figure 5A, B). It was observed that regulators associated with MSCs immunosuppressive function, such as RELB (https://doi.org/10.1182/blood.V116.21.4865.4865), and transcription-activating regulators, such as GAPB1 and EHF, were highly expressed in CD146^+^ NES^+^ MSCs.

### Building an interactive visualization application

To facilitate the exploration of single-cell transcriptomic data involved in this study, we created an interactive web application using the R package ShinyCell. This application allows researchers to interactively explore the expression patterns of different genes/regulators across various cell subpopulations. Furthermore, we packaged this web application into an executable desktop application using the Electron framework (Figure 5D). This desktop executable is compatible with both 32-bit and 64-bit Windows systems, requiring no installation or internet connectivity. It offers a wide range of visualization options, including scatter plots, violin plots, box plots, bubble plots, and more, enabling researchers to freely investigate the expression patterns of different genes/regulators across different cell subpopulations/sample sources. Currently, the desktop executable is available in the Mendeley database (https://data.mendeley.com/drafts/36tfjc42hm).

### Quantitative reverse transcription-polymerase chain reaction and Western blot

We sorted CD146^+^NES^+^ HUMSCs and CD146^−^NES^−^ HUMSCs by flow cytometry and then performed qPCR and Western blot analyses. In qPCR, we measured markers including RELB, ORC6, CCL2, RPL30, BUB3, FAU8, CXCL1, CDK1, CADD4, CXCL5, RPL39, and CXCL3, which were highly expressed in CD146^+^NES^+^ HUMSCs (Supplementary Figure S6A). Western blot analysis also revealed that the protein levels of RELB, FAU, and CXCL5 were higher in CD146^+^NES^+^ HUMSCs compared to CD146^−^NES^−^ HUMSCs (Supplementary Figure S6B).

## Discussion

Mesenchymal stem cells have been widely studied for their unique anti-inflammatory and immunomodulatory properties, making them a focus in medical research for treating various diseases, particularly complex and refractory conditions.^16-19^ Through paracrine functions, including secretion of certain cytokines and exosomes, MSCs not only exhibit direct anti-inflammatory and antimicrobial effects but also promote tissue regeneration, injury repair, anti-fibrosis, antioxidant stress, and anti-apoptosis mechanisms.^20,21^ However, the heterogeneity of MSCs, wherein they may exhibit different mechanisms of action when applied to specific diseases, poses a challenge to their clinical application.^22^

Evidence suggests a correlation between the biological functions of stem cells and the age of the donors. Studies have shown that compared to young individuals, adipose-derived MSCs (ASCs) from elderly individuals exhibit decreased migration and immunomodulatory capabilities.^23^ Additionally, MSCs undergo changes in epigenetics with increasing passages (aging), leading to decreased self-renewal and increased differentiation.^24^ Our previous research focused on HUMSCs from different gestational ages, demonstrating that preterm infants have advantages in cell proliferation and tissue injury repair capacity in vitro, indicating the presence of heterogeneity among them.^5^ Therefore, we are keen on exploring the intrinsic reasons for these differences, aiming to uncover their characteristics at the single-cell RNA transcriptome level.

Upon initial cell clustering and annotation, we observed that MSCs isolated from various tissues are not “uniform”; they comprise multiple cell subgroups, each potentially possessing distinct biological functions. Although most of these cell subgroups meet the criteria for MSCs at the RNA level, they may have different biological functions. Consequently, the heterogeneity observed in different tissues arises from the presence of distinct cell subgroups. Indeed, while the ISCT has established minimum criteria for defining MSCs, facilitating their widespread acquisition from tissues, this standardization has led to concerns about heterogeneity, prompting the need for stricter regulations to achieve homogeneity and improve clinical applications. Researchers have discovered that the expression of certain cell surface markers in MSCs may be associated with their biological properties. For instance, Stro-1-positive bone marrow MSCs are more effective in inhibiting PBMC proliferation, albeit their low yield poses a limitation in clinical use.^25^ Similarly, the absence of CD90 may be related to the loss of immunosuppressive potential in bone marrow MSCs.^26^ This surface marker appears to be involved in the control of soluble HLAG and IL-10 production, both of which participate in the immunosuppression process.^26^ CD271-positive bone marrow-derived MSCs also exhibit superior immunosuppressive abilities.^27^ Additionally, MSCs enriched with VCAM-1 have higher immunomodulatory activity,^28^ while MSCs double-positive for CD39 and CD73 appear to have potent inhibitory potential.^29^ It is speculated that most umbilical cord MSCs are mainly located around blood vessels in the Wharton’s jelly, suggesting that precursor cells of MSCs may migrate from perivascular tissues, implying that perivascular cells may be precursors of HUMSCs^30^ Perivascular cells express markers such as CD146, NG2, and PDGF-Rß, which contribute to the biological characteristics of MSCs.^4^ Therefore, we consider CD146^+^ HUMSCs as perivascular cells. Nestin is crucial for maintaining the self-renewal ability of stem cells and mesenchymal cells.^31-33^ Thus, we selected CD146 and Nestin as screening markers for our study.

CD146^+^ MSCs have been demonstrated to possess various unique properties in regenerative medicine, including homing ability, cell proliferation, and immunomodulation.^34,35^ This study also confirmed that CD146^+^ NES^−^ MSCs have advantages in cell proliferation, antimicrobial activity, immunomodulation, and low differentiation compared to CD146^−^ NES^−^ MSCs. However, they exhibit weaker biological functions in these aspects compared to CD146^+^ NES^+^ MSCs, indicating the significant role of Nestin. Originally discovered in neural stem cells, Nestin is widely present in stem and progenitor cells of other tissues, playing a crucial role in the self-renewal, migration, and differentiation of stem cells.^32^ While studies on Nestin-positive cells are more common in neural stem cells and bone marrow MSCs, research on Nestin in umbilical cord MSCs is limited. In our study, CD146^+^ NES^+^ MSCs were found to be highly expressed in preterm HUMSCs, whereas term HUMSCs mainly contained CD146^+^ NES^−^ MSCs. Our previous research suggested that preterm HUMSCs have advantages in cell proliferation and tissue repair compared to term HUMSCs. We speculate that this may be related to the presence of CD146^+^ NES^+^ MSCs. Although we also compared their differentiation potential, there was no significant advantage in differentiation toward osteogenic, chondrogenic, adipogenic, vascular, and neural lineages compared to Nestin-negative MSCs. However, compared to Nestin-negative MSCs, CD146^+^ NES^+^ MSCs exhibited a lower degree of cell differentiation, indicating that they may be more prominent in cell pluripotency. Although the multipotency of MSCs has been questioned, with a growing consensus on their paracrine functions in tissue regeneration,^36-38^ there may be less attention paid to the existence of cells like CD146^+^ NES^+^ MSCs. We eagerly anticipate the involvement of more researchers in this area of research in the near future. In our study, several key immunomodulatory-related transcripts, including RELB, GAPB1, and EHF, were found to be highly expressed in CD146^+^ NES^+^ MSCs. Among them, RelB, a member of the NF-κB/Rel family, plays a dominant role in the non-canonical NF-κB pathway.^39^ During the inflammatory response, RelB can inhibit the expression of proinflammatory factors such as IL-1β and TNF-α, while promoting the expression of anti-inflammatory factor IKBα^.40-42^ This may be one of the reasons why CD146^+^ NES^+^ MSCs have an advantage in antimicrobial and immunomodulatory functions.

Our study has several limitations. We solely analyzed CD146^+^ NES^+^ MSCs at the single-cell RNA transcription level. CD146 is a surface antigen and can be directly sorted using flow cytometry. However, Nestin is expressed in the cytoplasm, so it cannot be directly sorted.^43^ The cell membrane needs to be permeabilized to allow antibodies to enter the cells. Therefore, we used a permeabilization method for flow cytometry in this study. After sorting, the cells were used directly for RNA extraction and protein analysis and could not be further cultured or expanded in vitro.To address this issue, we conducted a literature search and found studies that sort cells using surface markers closely related to Nestin, followed by in vitro expansion. These markers include CD29^+^CD51^+^ and PDGFRα^+^CD51^+^.^44,45^ Therefore, in our future work, we plan to use a similar approach to sort double-positive cells from HUMSCs, expand them in vitro, and assess their stability, and thoroughly analyze their biological characteristics. Finally, we will validate their safety and efficacy in specific disease animal models.

Additionally, MSCs derived from pluripotent stem cells have shown many advantages in cell proliferation, antimicrobial activity, and immunomodulation.^46-48^ GMP-grade MSCs derived from pluripotent stem cells have been used in clinical trials for refractory graft-versus-host disease (GVHD).^49,50^ Previous studies have found that the efficiency of obtaining iPS cells is closely related to cell morphology, proliferation levels, and the expression of endogenous factors.^51^ Therefore, it is worth further investigating whether iPSCs derived from CD146^+^NES^+^ HUMSCs have any advantages in these aspects. In conclusion, we have generated the single-cell transcriptome profiles of HUMSCs at 22 + 5, 28, and 39 weeks, and compared them with publicly available single-cell data of bone marrow MSCs. We have dissected the distribution and biological characteristics of CD146^+^ NES^+^ MSCs in MSCs from different tissue sources at the single-cell level, elucidating their advantages in cell proliferation, antimicrobial activity, immunomodulation, and low differentiation, along with their potential molecular mechanisms. This provides new insights and a scientific basis for the future clinical application of CD146^+^ NES^+^ MSCs.

## Supplementary Material

sxae063_suppl_Supplementary_Figure_S1

sxae063_suppl_Supplementary_Figure_S2

sxae063_suppl_Supplementary_Figure_S3

sxae063_suppl_Supplementary_Figure_S4

sxae063_suppl_Supplementary_Figure_S5

sxae063_suppl_Supplementary_Figure_S6

sxae063_suppl_Supplementary_Table_S1

sxae063_suppl_Supplementary_Table_S2

sxae063_suppl_Supplementary_Table_S3

sxae063_suppl_Supplementary_Table_S4

sxae063_suppl_Supplementary_Table_S5

## Data Availability

Additional tables and high-resolution plots are included in Supplementary Materials. The single-cell transcriptome data of HUMSCs (22 + 5, 28W) are shared in Mendeley Data (https://data.mendeley.com/drafts/36tfjc42hm).
