# Supplementary material for: Advantages of cell proliferation and immune regulation in CD146+NESTIN+ HUMSCs: insights from single-cell RNA sequencing
Source: Stem Cells. 2024 Oct 21;43(7):sxae063. doi: 10.1093/stmcls/sxae063 (PMC12199618; doi:10.1093/stmcls/sxae063)
Supplement: sxae063_suppl_Supplementary_Figure_S1 [file sxae063_suppl_supplementary_figure_s1.pdf]

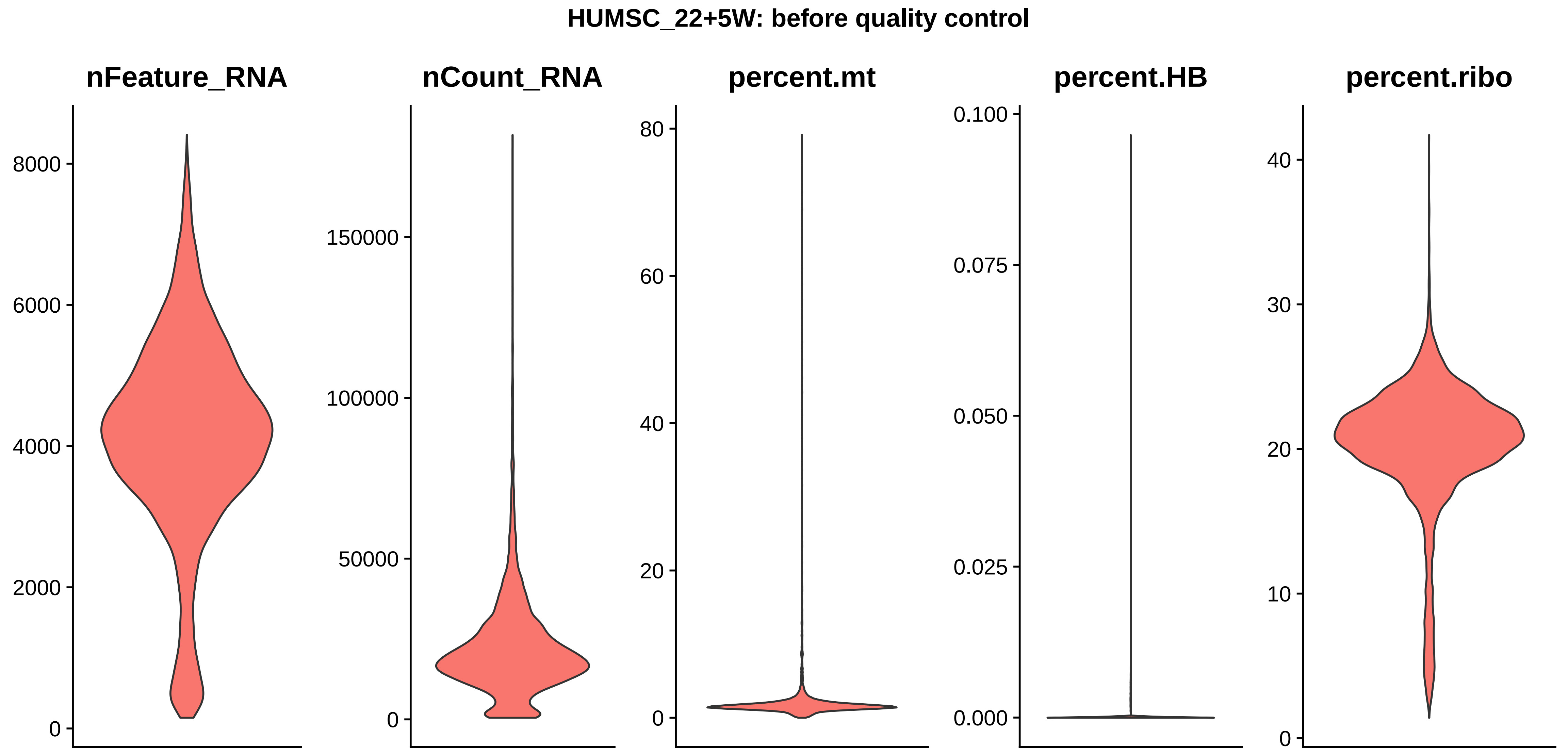

HUMSC_22+5W: before quality control
nFeature_RNA
nCount_RNA
percent.mt
percent.HB
percent.ribo
0
2000
4000
6000
8000
0
50000
100000
150000
0
20
40
60
80
0.000
0.025
0.050
0.075
0.100
0
10
20
30
40

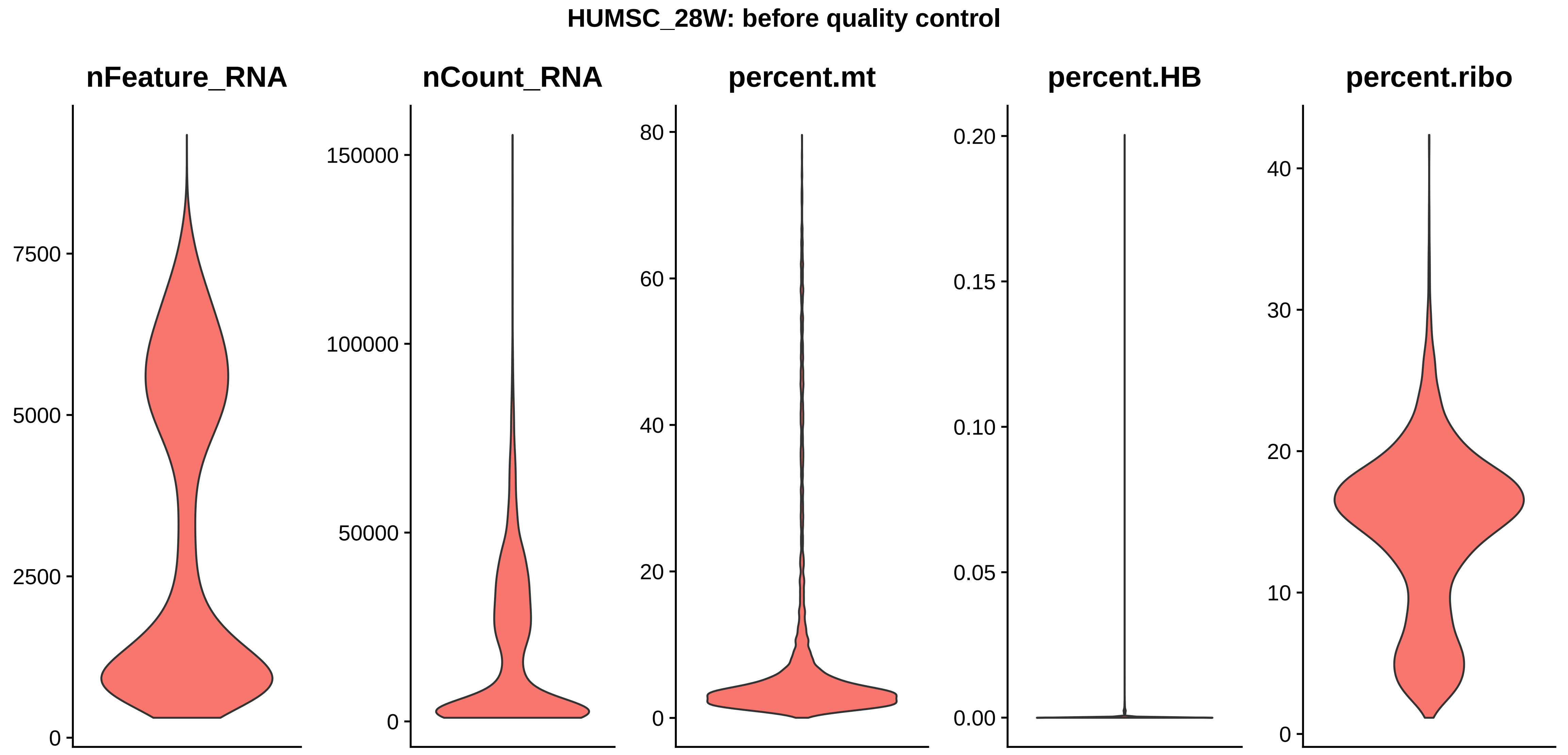

HUMSC_28W: before quality control
nFeature_RNA
nCount_RNA
percent.mt
percent.HB
percent.ribo
0
2500
5000
7500
0
50000
100000
150000
0
20
40
60
80
0.00
0.05
0.10
0.15
0.20
0
10
20
30
40

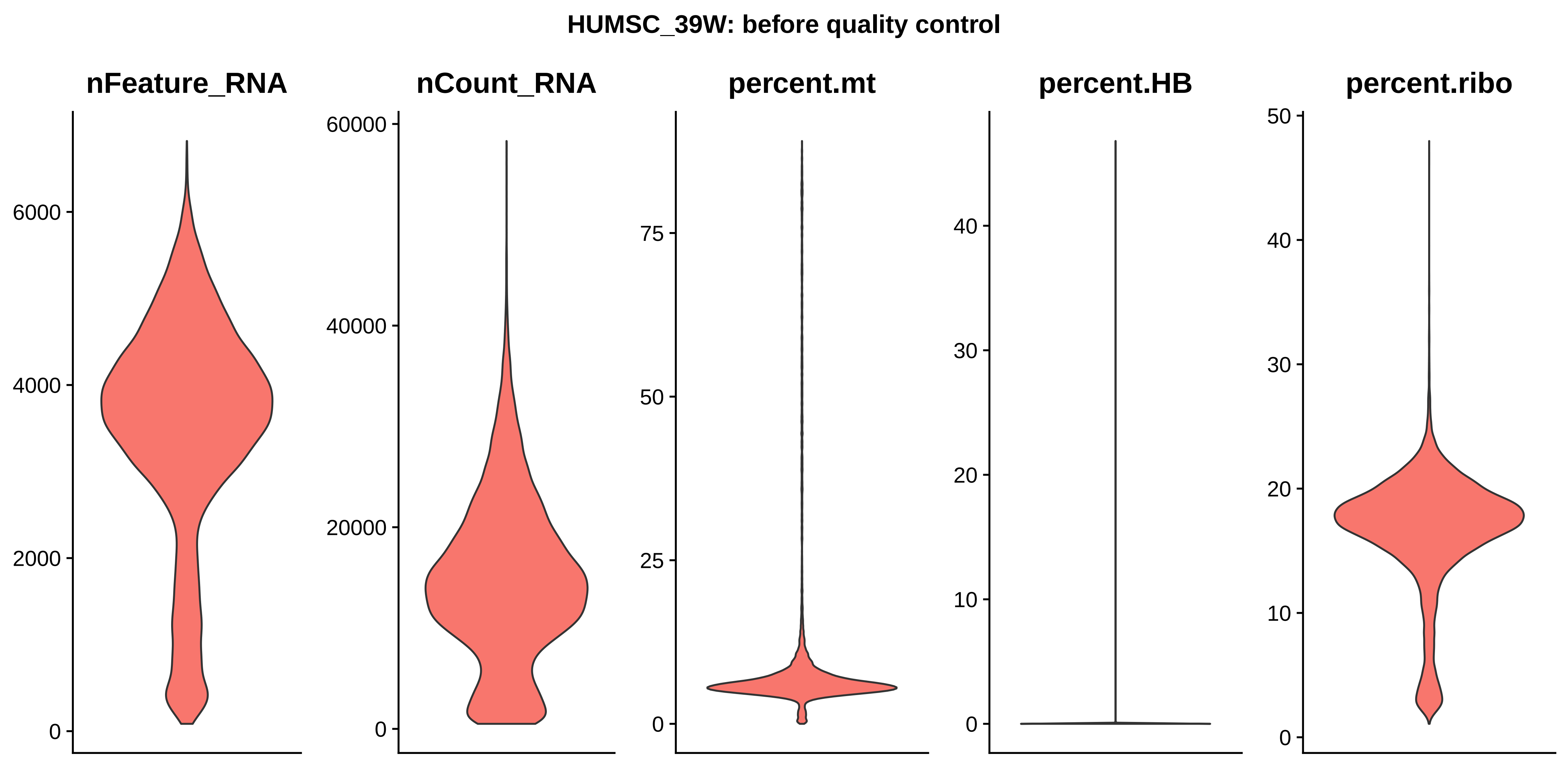

HUMSC_39W: before quality control
nFeature_RNA
nCount_RNA
percent.mt
percent.HB
percent.ribo
0
2000
4000
6000
0
20000
40000
60000
0
25
50
75
0
10
20
30
40
0
10
20
30
40
50

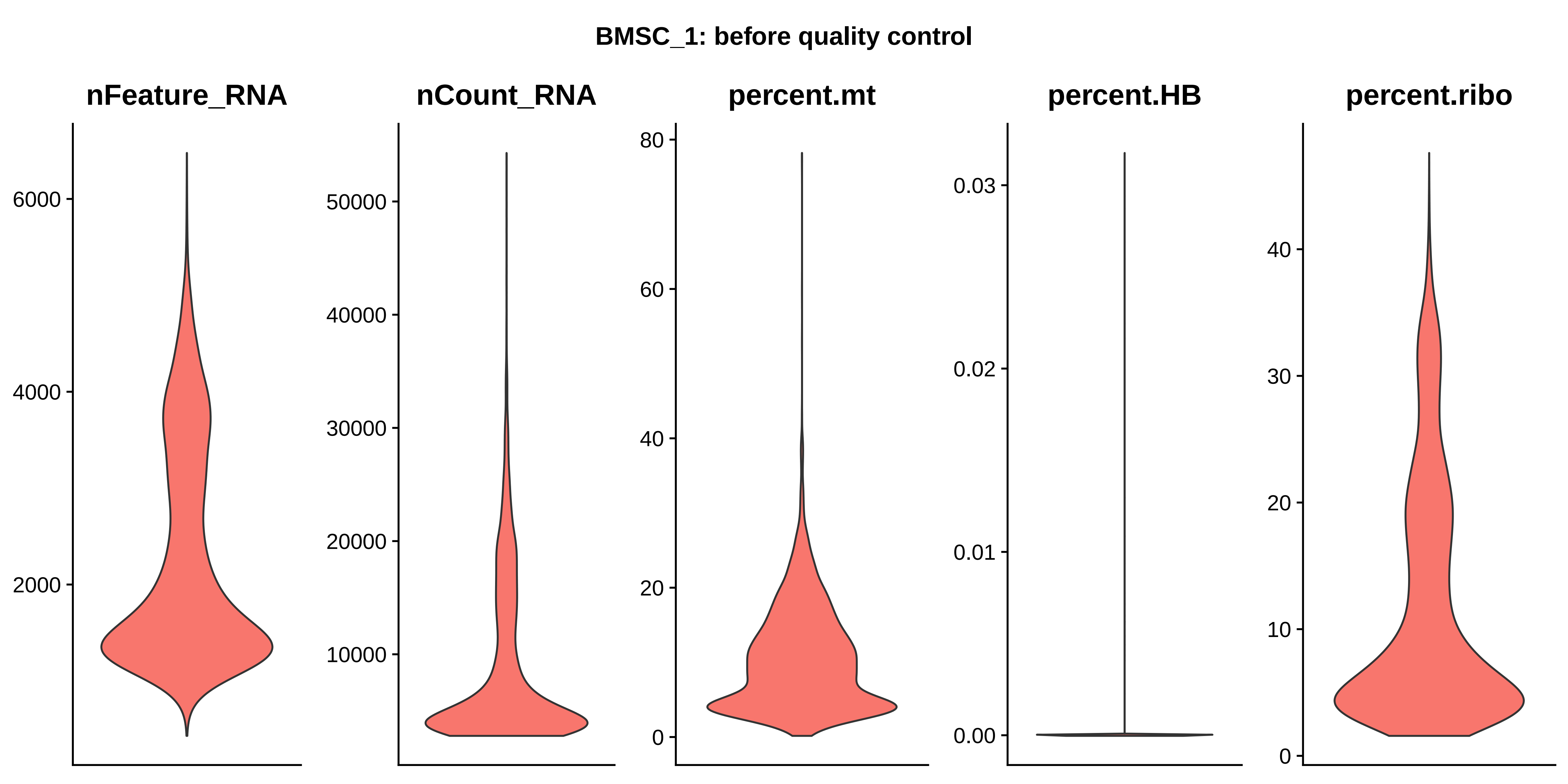
BMSC_1: before quality control
nFeature_RNA
nCount_RNA
percent.mt
percent.HB
percent.ribo
6000
4000
2000
50000
40000
30000
20000
10000
80
60
40
20
0
0.03
0.02
0.01
0.00
40
30
20
10
0

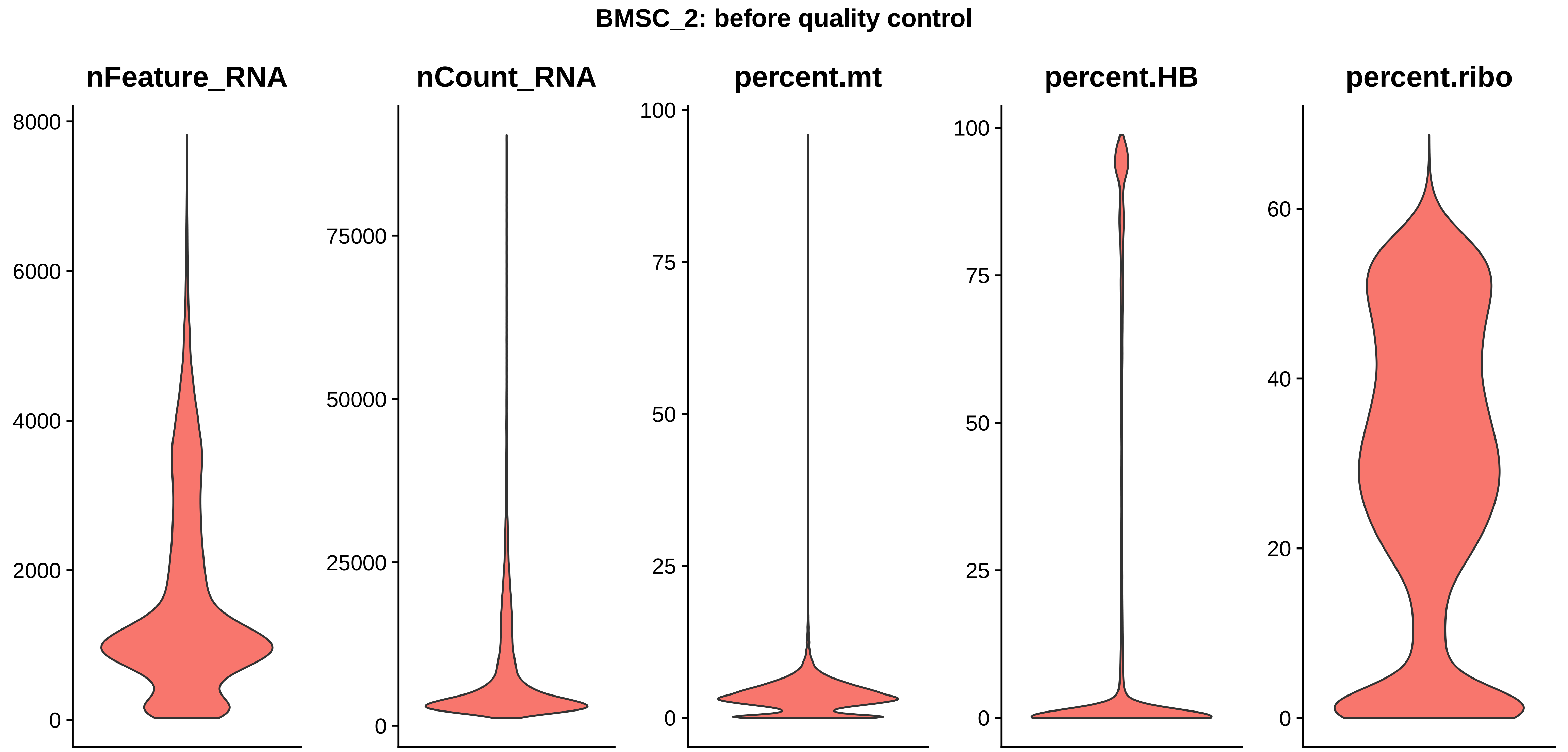
BMSC_2: before quality control
nFeature_RNA
nCount_RNA
percent.mt
percent.HB
percent.ribo
8000
6000
4000
2000
0
75000
50000
25000
0
100
75
50
25
0
100
75
50
25
0
60
40
20
0
