# Supplementary material for: Advantages of cell proliferation and immune regulation in CD146+NESTIN+ HUMSCs: insights from single-cell RNA sequencing
Source: Stem Cells. 2024 Oct 21;43(7):sxae063. doi: 10.1093/stmcls/sxae063 (PMC12199618; doi:10.1093/stmcls/sxae063)
Supplement: sxae063_suppl_Supplementary_Figure_S2 [file sxae063_suppl_supplementary_figure_s2.pdf]

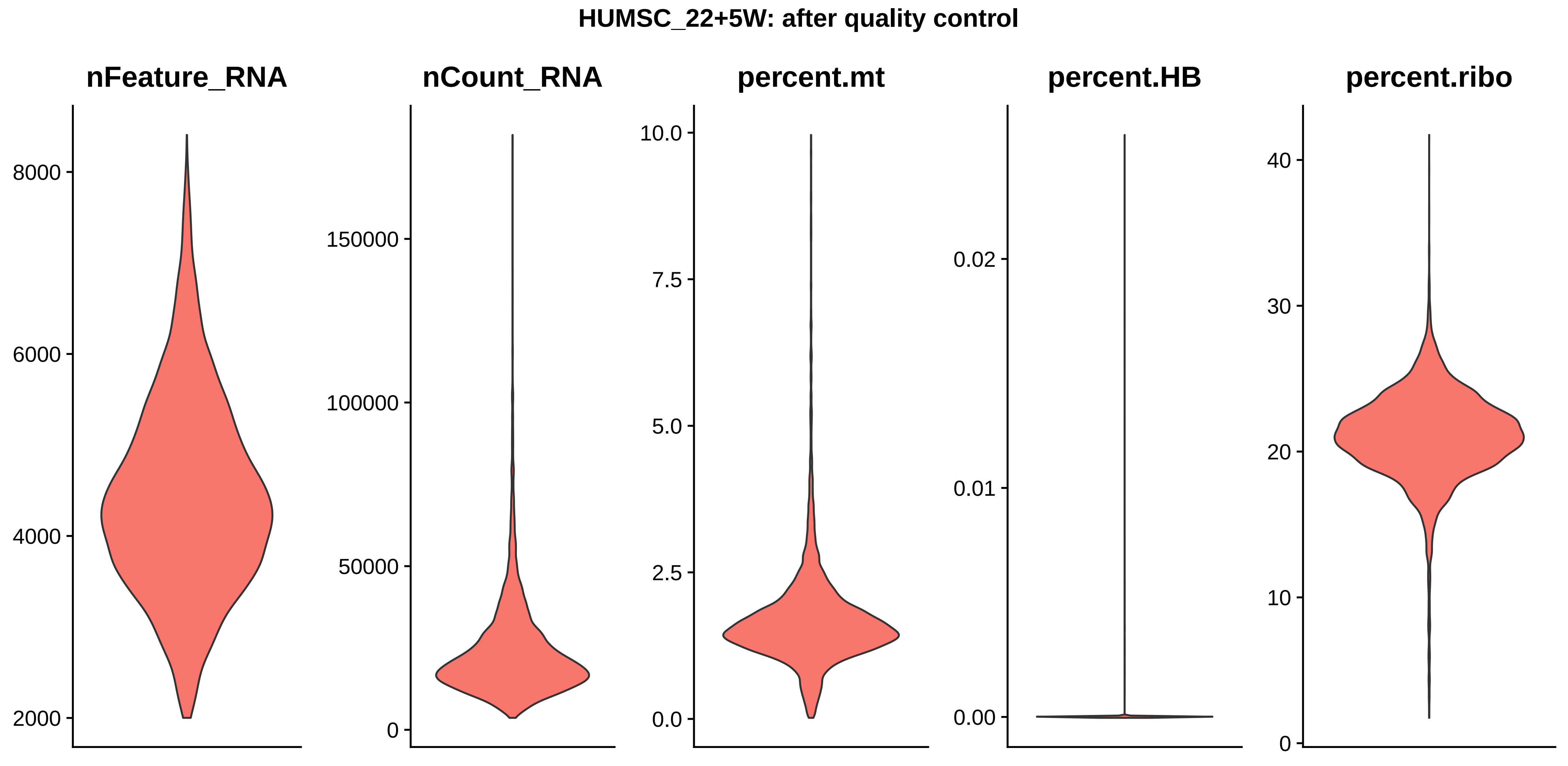
HUMSC_22+5W: after quality control
nFeature_RNA
nCount_RNA
percent.mt
percent.HB
percent.ribo
8000
6000
4000
2000
150000
100000
50000
0
10.0
7.5
5.0
2.5
0.0
0.02
0.01
0.00
40
30
20
10
0

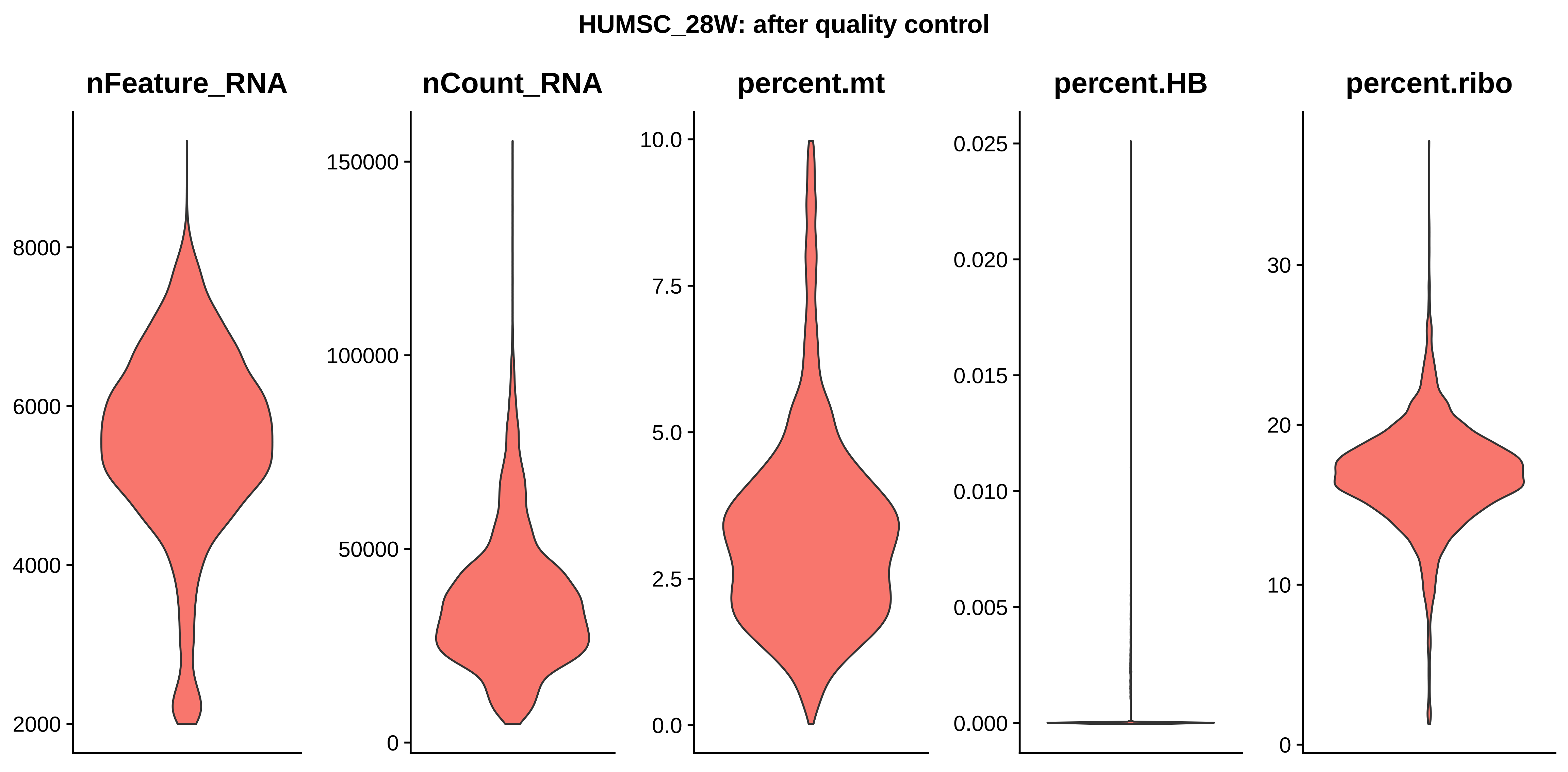
HUMSC_28W: after quality control
nFeature_RNA
nCount_RNA
percent.mt
percent.HB
percent.ribo
2000
4000
6000
8000
0
50000
100000
150000
0.0
2.5
5.0
7.5
10.0
0.000
0.005
0.010
0.015
0.020
0.025
0
10
20
30

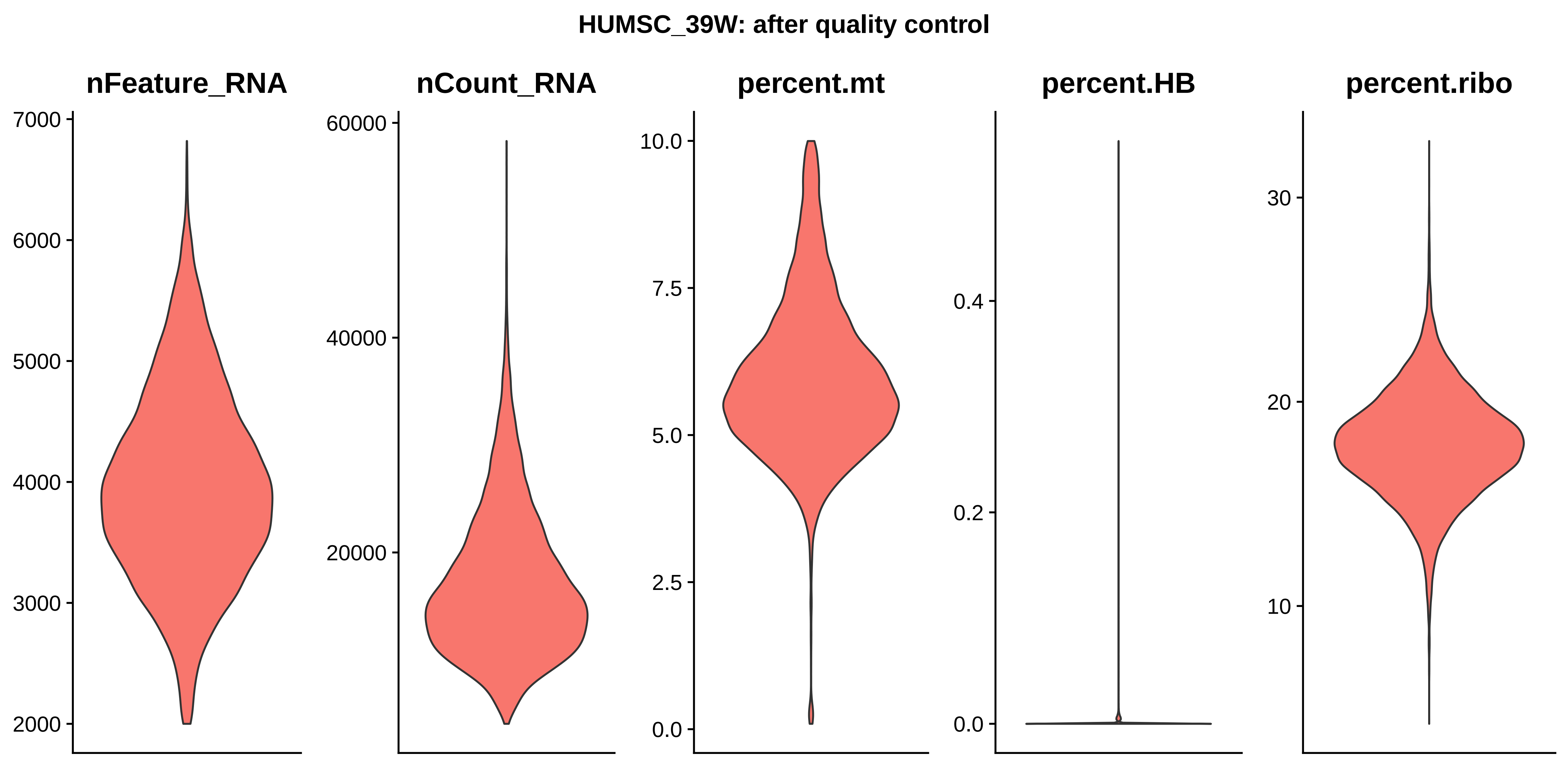

HUMSC_39W: after quality control
nFeature_RNA
nCount_RNA
percent.mt
percent.HB
percent.ribo
7000
6000
5000
4000
3000
2000
60000
40000
20000
10.0
7.5
5.0
2.5
0.0
0.4
0.2
0.0
30
20
10

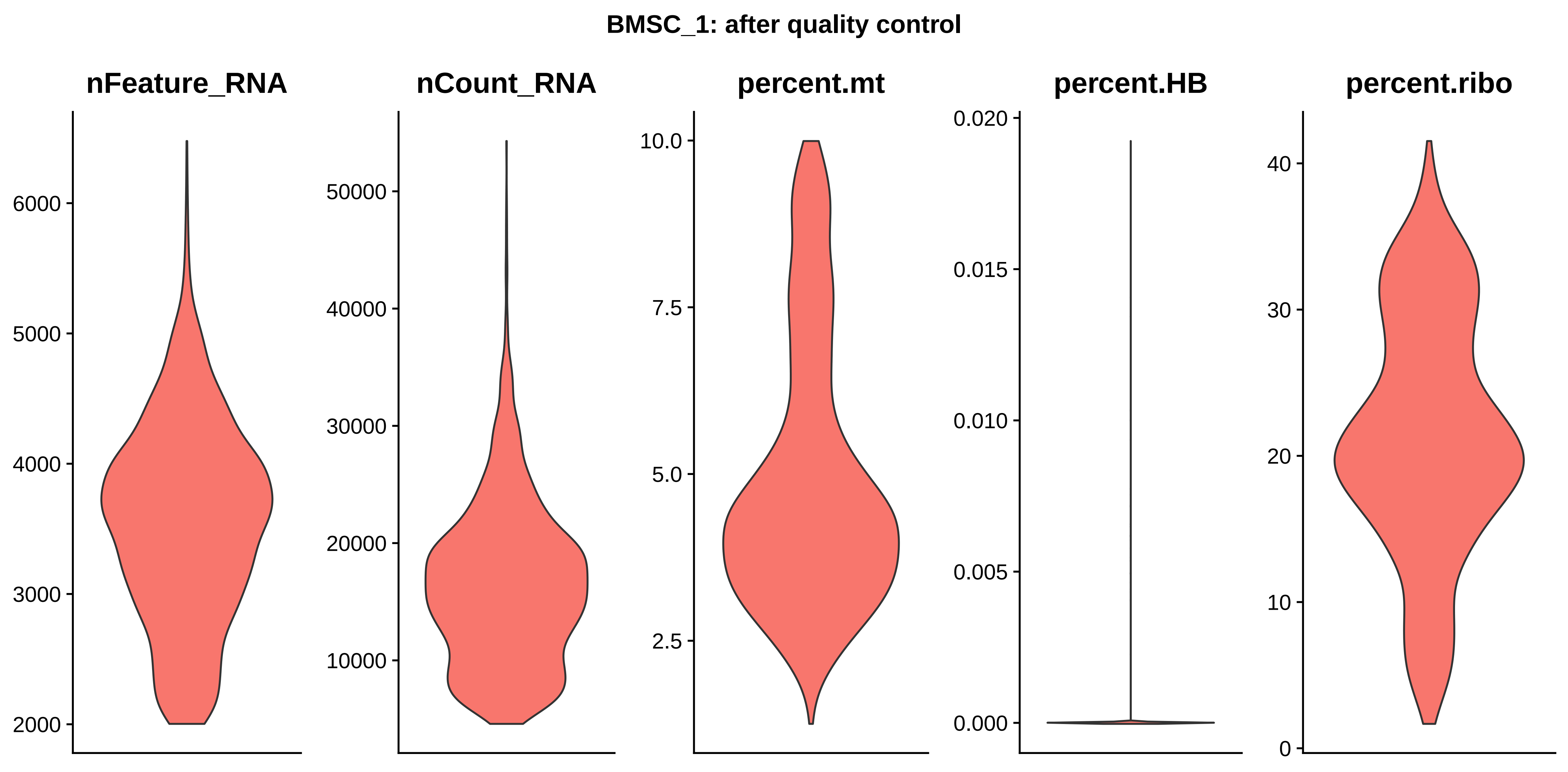

BMSC_1: after quality control
nFeature_RNA
nCount_RNA
percent.mt
percent.HB
percent.ribo
2000
3000
4000
5000
6000
10000
20000
30000
40000
50000
2.5
5.0
7.5
10.0
0.000
0.005
0.010
0.015
0.020
0
10
20
30
40

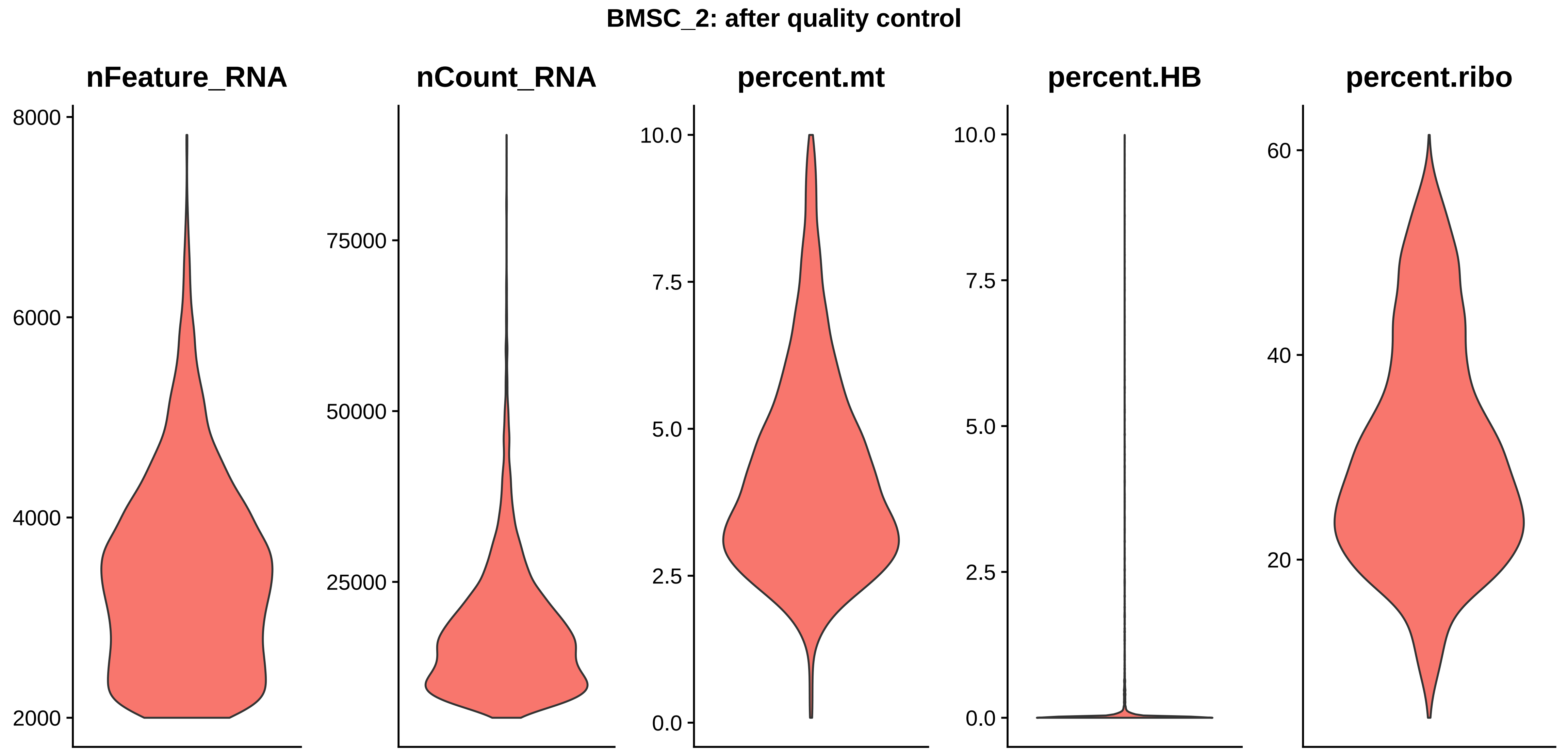

BMSC_2: after quality control
nFeature_RNA
nCount_RNA
percent.mt
percent.HB
percent.ribo
8000
6000
4000
2000
75000
50000
25000
10.0
7.5
5.0
2.5
0.0
10.0
7.5
5.0
2.5
0.0
60
40
20
