# Supplementary figures and images for: Advantages of cell proliferation and immune regulation in CD146+NESTIN+ HUMSCs: insights from single-cell RNA sequencing
Source: Stem Cells. 2024 Oct 21;43(7):sxae063. doi: 10.1093/stmcls/sxae063 (PMC12199618; doi:10.1093/stmcls/sxae063)

PCA

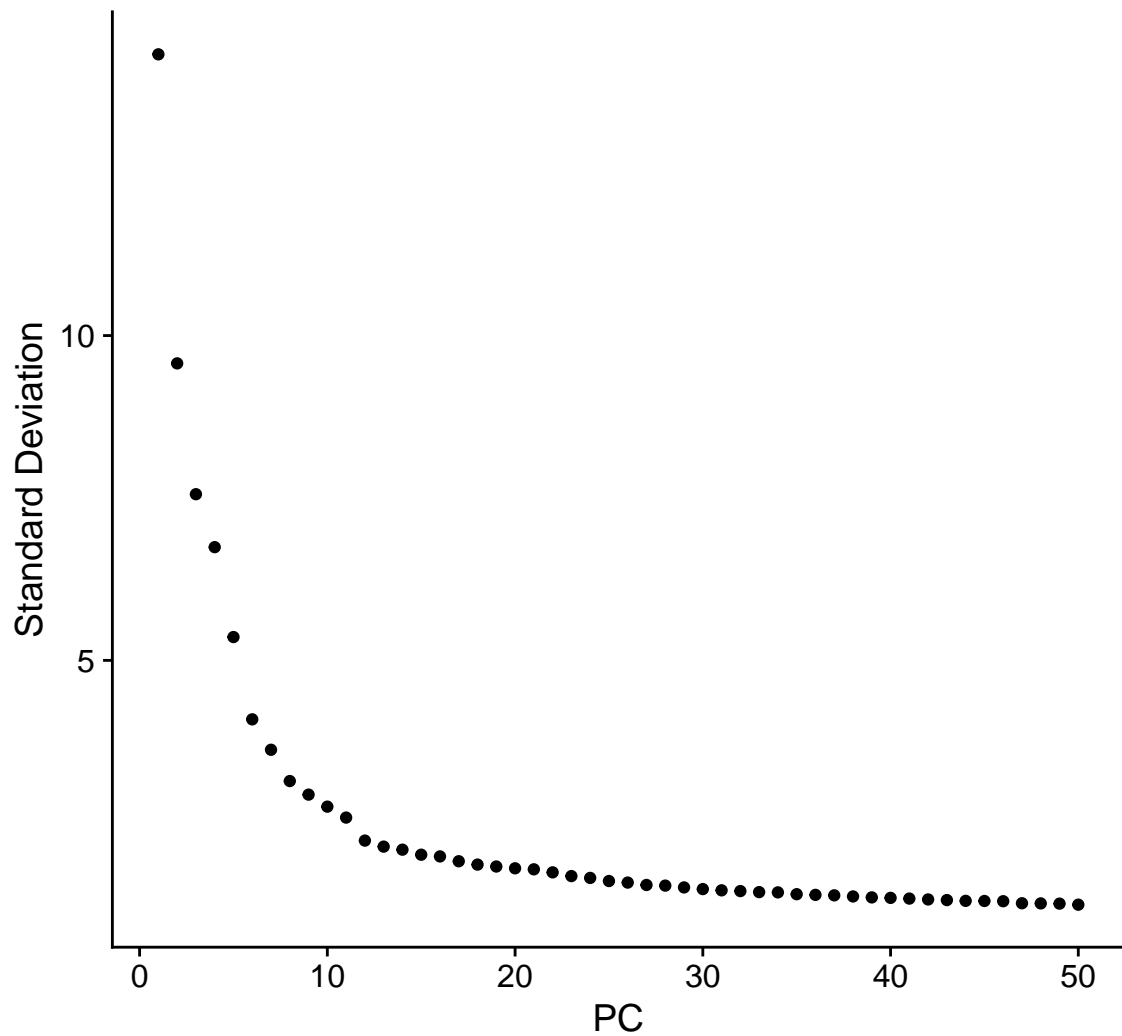

Harmony

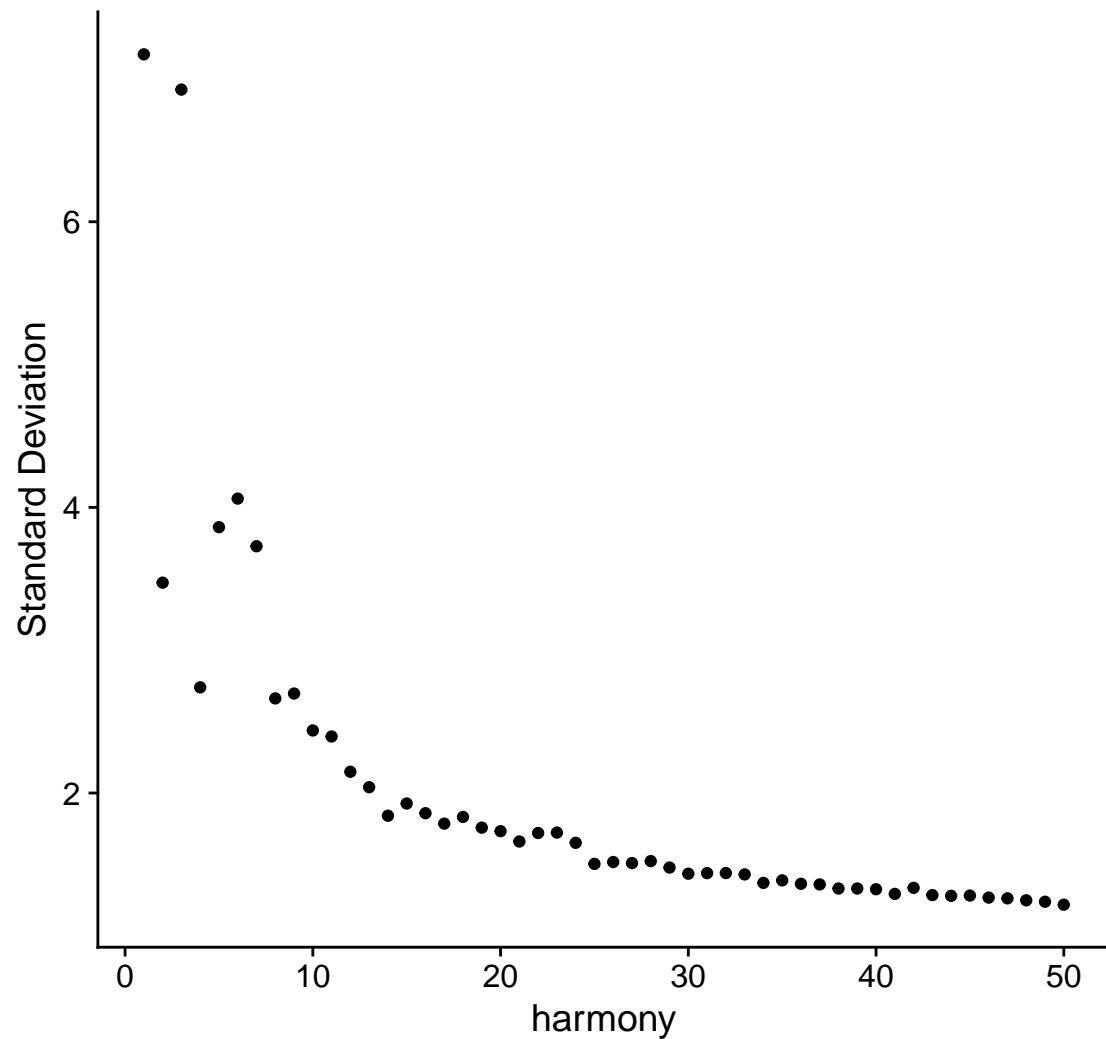

Supplement: sxae063_suppl_Supplementary_Figure_S3 [file sxae063_suppl_supplementary_figure_s3.pdf]

Dendrogram

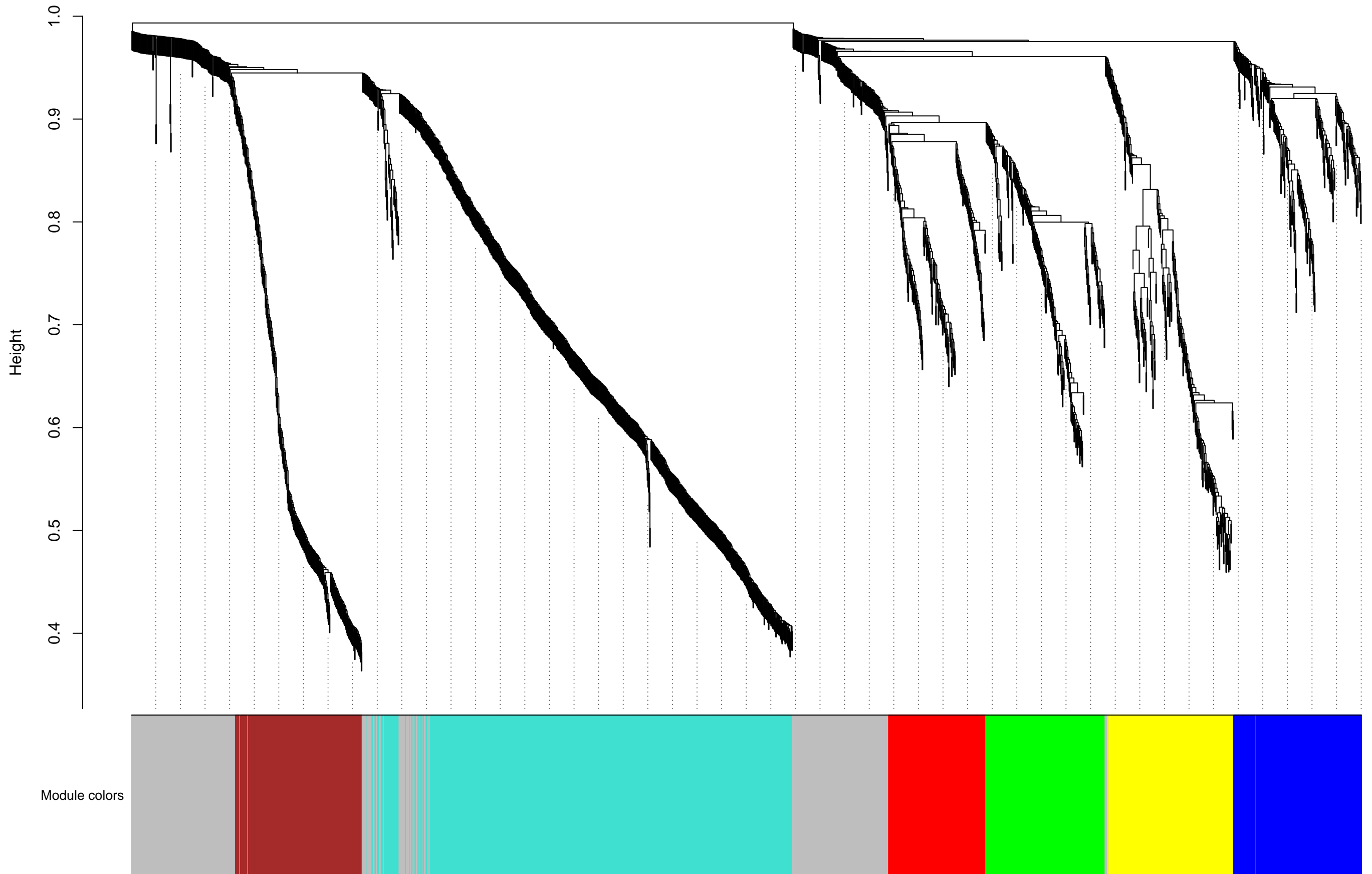

Supplement: sxae063_suppl_Supplementary_Figure_S4 [file sxae063_suppl_supplementary_figure_s4.pdf]

A

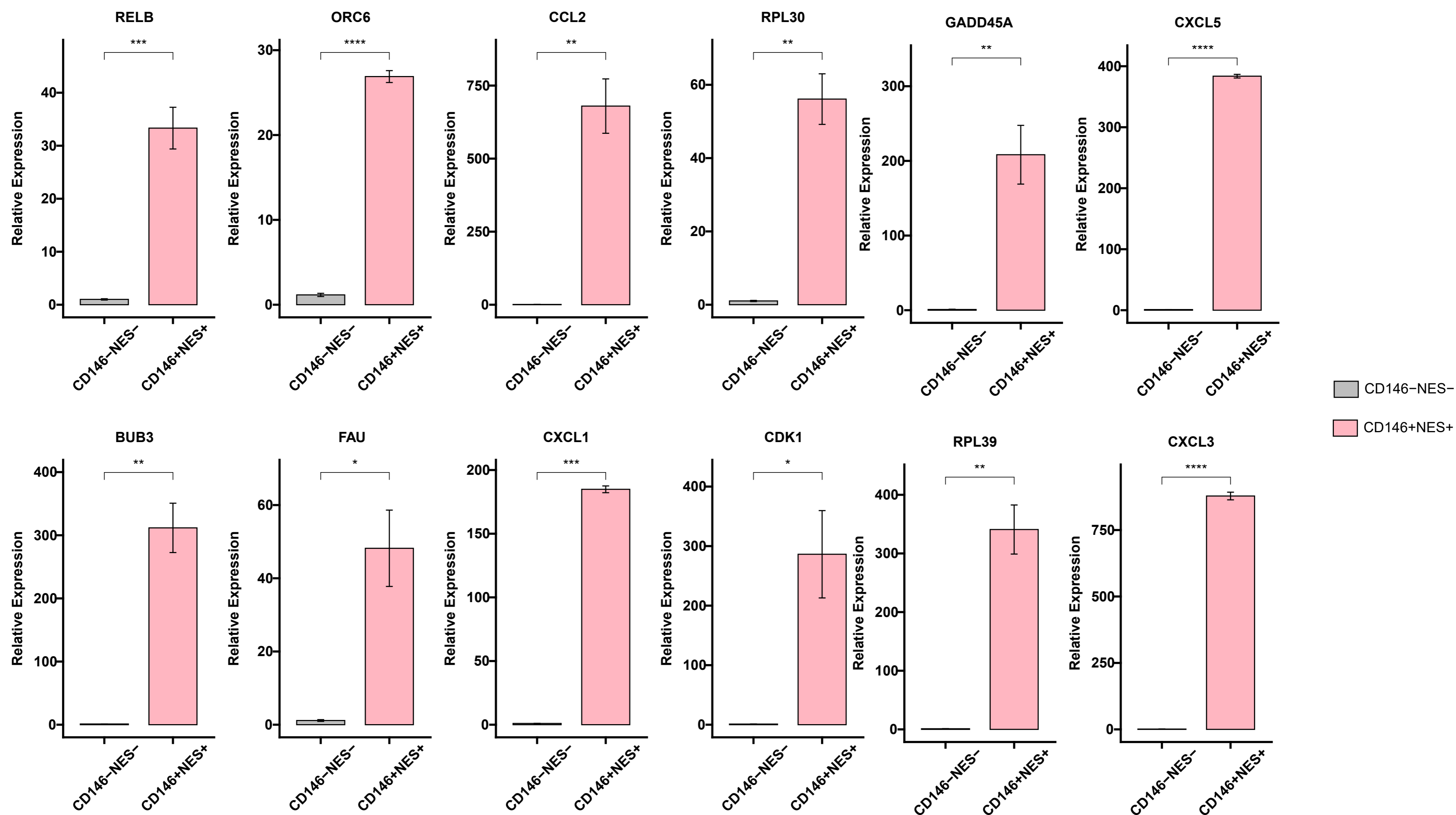

B

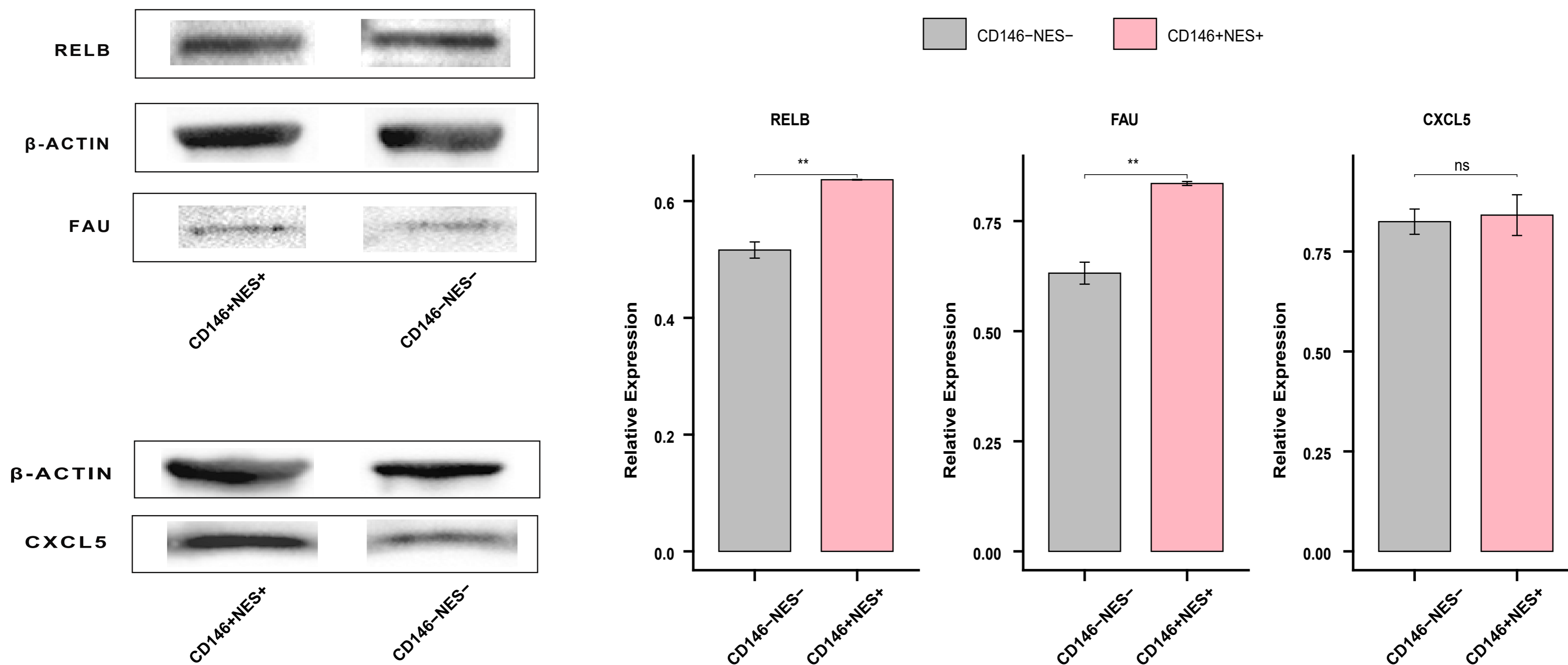

Supplement: sxae063_suppl_Supplementary_Figure_S5 [file sxae063_suppl_supplementary_figure_s5.pdf]
